# Conserved sleep disturbances in FOXP1 syndrome originate from developmental dysregulation of peptidergic signaling

**DOI:** 10.1172/JCI193475

**Published:** 2026-04-01

**Authors:** Mireia Coll-Tané, Ilse Eidhof, Jie Han, Nicholas Raun, Lara V. van Renssen, Simon E. Fisher, Matthew S. Kayser, Tjitske Kleefstra, Sigrid Pillen, Caitlin M. Hudac, Jordi Mayneris-Perxachs, Marieke Klein, Saskia Koene, Anna Castells-Nobau, Annette Schenck

**Affiliations:** 1 Department of Human Genetics and; 2 Donders Institute for Brain, Cognition, and Behaviour, Radboud University Medical Center, Nijmegen, Netherlands.; 3 Language and Genetics Department, Max Planck Institute for Psycholinguistics, Nijmegen, Netherlands.; 4 Donders Centre for Neuroscience, Donders Institute for Brain, Cognition, and Behaviour, Radboud University, Nijmegen, Netherlands.; 5 Departments of Psychiatry and Neuroscience, Chronobiology and Sleep Institute, Perelman School of Medicine at the University of Pennsylvania, Philadelphia, Pennsylvania, USA.; 6 Department of Clinical Genetics, Erasmus University Rotterdam, Rotterdam, Netherlands.; 7 Center of Excellence for Neuropsychiatry, Vincent van Gogh Institute for Psychiatry, Venray, Netherlands.; 8 Kinderslaapexpert BV (Pediatric Sleep Expert Ltd.), Mook, Netherlands.; 9 Department of Psychology and; 10 Carolina Autism and Neurodevelopment Research Center, University of South Carolina, Columbia, South Carolina, USA.; 11 Nutrition, Eumetabolism and Health Group and Integrative Systems Medicine and Biology Group, Girona Biomedical Research Institute (IDIBGI-CERCA), Girona, Spain.; 12 Centro de Investigación Biomédica en Red de Fisiopatología de la Obesidad y Nutrición (CIBEROBN), Madrid, Spain.; 13 Department of Medical Neuroscience, Radboud University Medical Center, Nijmegen, Netherlands.

**Keywords:** Clinical Research, Neuroscience, Behavior, Complex traits, Genetic diseases

## Abstract

Sleep disturbances are among the most prevalent clinical features of FOXP1 syndrome, yet their nature and underlying mechanisms remain unclear. Here, we report that individuals with FOXP1 syndrome suffer from insomnia with sleep maintenance problems and early waking. Consistently, common variants in *FOXP* genes were associated with insomnia symptoms and short sleep. These sleep disturbances were recapitulated in *Drosophila* *FoxP* mutants, which exhibit severely fragmented and reduced sleep. *FoxP* loss also led to circadian arrhythmicity and impaired the plasticity of neuropeptide pigment dispersing factor–secreting (PDF-secreting) neurons in a non-cell-autonomous manner. *FoxP* was required during development for adult sleep integrity, particularly in peptidergic neurons. Transcriptomic analyses revealed a dysregulation of genes involved in peptidergic signaling, including *hugin*. *FoxP* was expressed in hugin^+^ neurons (afferent to PDF-secreting neurons) during development, and its knockdown in these cells was sufficient to induce sleep fragmentation. Our findings establish an evolutionarily conserved role for FOXP proteins in the peptidergic regulation of sleep.

## Abstract 2

FOXP1 syndrome leads to specific sleep problems, which are also seen in fruit flies, due to problems with peptidergic signaling during brain development.

## Introduction

Sleep disturbances are among the most common co-occurring features of neurodevelopmental disorders (NDDs), affecting up to 86% of individuals compared with approximately 20% of typically developing children (1). These disturbances substantially impact the quality of life of both patients and their families, often posing a greater burden than other physical or cognitive problems (1, 2). Although effective therapies exist for sleep disorders in the typically developing population, and despite evidence that sleep interventions can improve behavioral and cognitive outcomes (3), such approaches have not been widely implemented for individuals with NDDs. This is largely due to the long-standing perception that sleep disturbances in these individuals are secondary consequences rather than core phenotypes of their syndromes (4), leading to the assumption that they are refractory to therapy. As a result, the underlying mechanisms of sleep dysfunction in NDDs remain poorly understood and understudied. We propose that monogenic neurodevelopmental syndromes characterized by highly prevalent and specific sleep disturbances offer a unique opportunity to investigate disease pathophysiology, including the mechanisms driving sleep dysfunction (5–7). At the same time, studying these syndromes has the potential to yield fundamental insights into the biology of sleep and its mechanistic links to cognitive disorders.

FOXP1 syndrome is a monogenic neurodevelopmental syndrome caused by pathogenic variants in the *forkhead box P1* (*FOXP1*) gene (OMIM #613670), which encodes a key transcription factor implicated in nervous system development (8). This syndrome is characterized by intellectual disability and/or global developmental delay, speech delay and articulation difficulties, autistic features or autism spectrum disorder (ASD) diagnosis, and mild dysmorphic traits (9, 10). Sleep disturbances in FOXP1 syndrome have long been overlooked and underreported, with early estimates suggesting that only about 5% of affected individuals experience sleep difficulties (9), a figure unrealistically lower than the approximately 20% prevalence observed in neurotypical children (2, 11). However, a recent comprehensive questionnaire assessing the medical signs, symptoms, and most burdensome complaints of FOXP1 syndrome identified sleep problems as one of the most prevalent clinical features, affecting 45% of individuals (12). This percentage resembles the recently reported prevalence of sleep disturbances (42%) in a cohort with pathogenic variants in *FOXP2*, another closely related member of the *FOXP* transcription factor family (13). Moreover, pathogenic variants in *FOXP4* cause a distinct NDD syndrome with overlapping clinical features (14). It is thus conceivable that *FOXP1*, *FOXP2*, and potentially *FOXP4*, which homo- and heterodimerize, control sleep through regulation of shared target genes in brain regions where they are coexpressed (15, 16). However, at present, sleep problems in FOXP1 syndrome are uncharacterized, and it remains unknown whether and how FOXP family proteins play a specific role in regulating sleep.

In this cross-species and cross-disciplinary study, we integrated analysis of sleep in individuals with FOXP1 syndrome with analysis of common variants in *FOXP1* and paralogs (*FOXP2* and *FOXP4*) that impact sleep traits in the general population to assess their role in sleep regulation. We further utilized an animal model, the fruit fly *Drosophila melanogaster*, to dissect the developmental, cellular, and molecular mechanisms by which pathogenic variants in *FOXP* genes lead to the disruption of sleep disturbances. Our work uncovers a novel, evolutionarily conserved role for the *FOXP* transcription factor family in sleep regulation.

## Results

### Individuals with pathogenic variants in FOXP1 suffer from sleep disturbances.

In a recent parent-report questionnaire study in a cohort with FOXP1 syndrome, sleep problems emerged as a highly reported symptom with a 45% prevalence (12). To obtain a first indication of the potential nature of these disturbances, we identified 6 individuals with likely gene-disruptive mutations (LGDMs) in *FOXP1* (genetic diagnosis detailed in Supplemental Table 1; supplemental material available online with this article; https://doi.org/10.1172/JCI193475DS1) from 2 large studies of NDD cohorts characterized by rare ASD-associated single-gene etiologies: the Simons Simplex Collection (SSC) (17, 18) and the National Institute of Mental Health–funded study (19). We extracted all available annotated sleep-related data for these individuals.

Among the 6 individuals with LGDMs in *FOXP1*, 4 (67%) reported frequent night awakenings, 3 (50%) experienced sleep-onset related issues, and 3 (50%) had daytime complaints (Table 1 and Supplemental Table 1). Due to the absence of an independent, age-matched neurotypical control group that underwent comparable genetic and phenotypic assessments, we compared these rates to 2 reference groups from the SSC: (a) a cohort with idiopathic ASD (i.e., ASD without a known genetic etiology) and (b) a cohort with NDDs caused by LGDMs in genes other than *FOXP1*. The prevalence of sleep complaints in individuals with *FOXP1* mutations was broadly similar to the idiopathic ASD cohort (*n* = 2509, *P* = 0.74) and in the cohort with LGDMs in other genes (*n* = 372, *P* > 0.99). Across development, older children were more likely to have sleep onset issues, though a trend suggests this effect was stronger for FOXP1 (Spearman’s ρ = 0.89, *P* = 0.017) than for those with idiopathic ASD [Spearman’s ρ = 0.06, *P* = 0.004; F(1, 2,266) = 3.21, *P* = 0.073].

**Figure 1:**
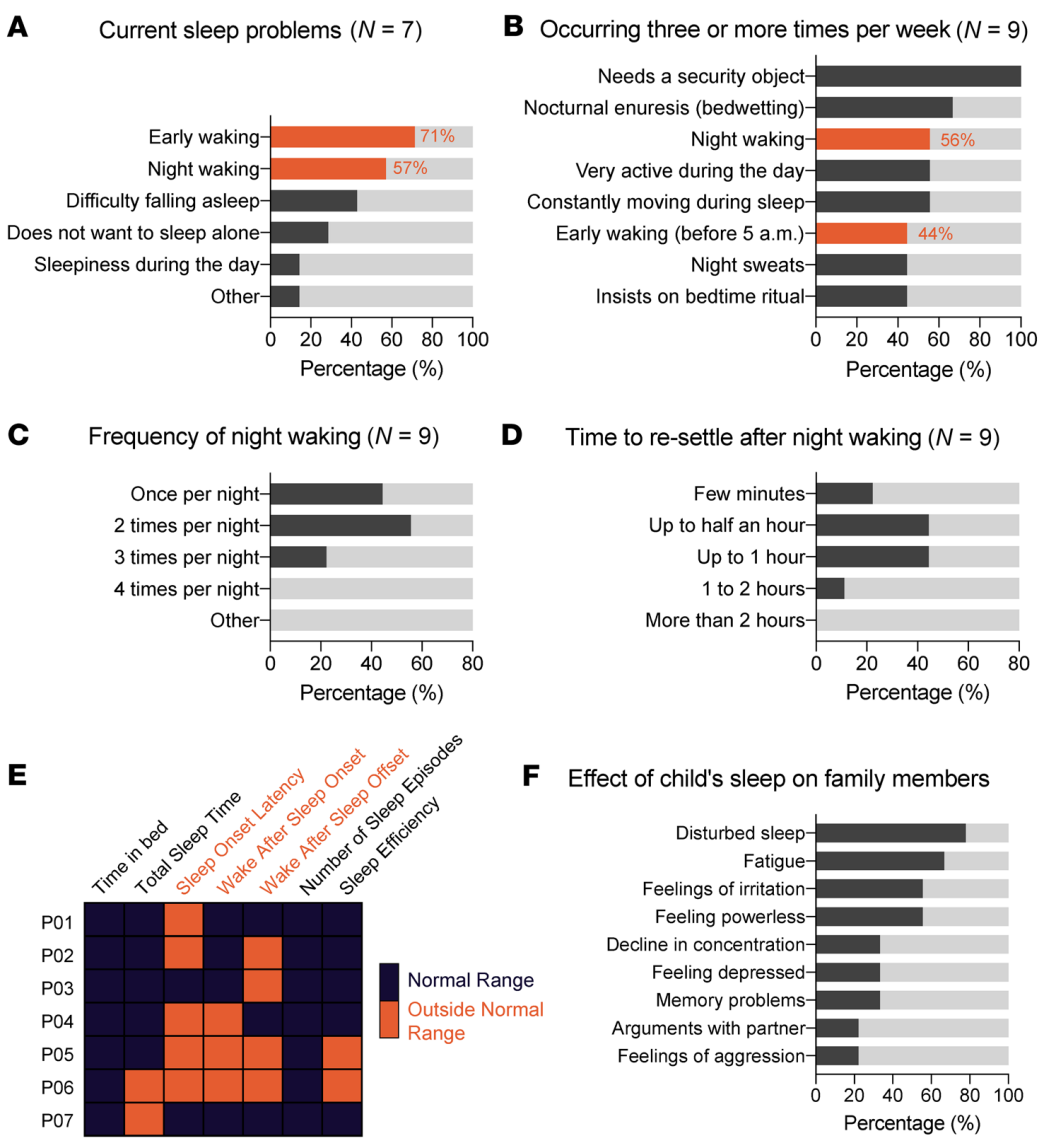
Individuals with FOXP1 syndrome suffer from night and early wakings that affect their family’s well-being. (**A**) Nature of the sleep problems reported by the parents of individuals with FOXP1 syndrome who believe their child currently has disturbed sleep (7 out of 9 individuals). (**B**) Sleep disturbances reported to occur at least 3 times per week in the whole cohort (*n* = 9), independently of whether the parents consider that they are currently experiencing sleep problems. Sleep complaints occurring 3 or more times per week are defined as a sleep disorder according to the ICSD-3 criteria (23). Only features occurring in at least 40% of our cohort are reported. (**C** and **D**) Frequency of night wakings (**C**) and estimated time taken to resettle after waking during the night (**D**) in our FOXP1 syndrome cohort (*n* = 9). (**E**) Outcome of the quantitative graphical sleep diaries. Quantitative values outside the range of neurotypical individuals (age considered; see Supplemental Methods) are highlighted in orange. Numerical values are provided in Table 2. (**F**) Effect of child’s sleep on family members (*n* = 9).

In addition to these available yet limiting data, we collected qualitative and/or quantitative sleep data from 9 individuals who reported sleep problems in a previous parent-report questionnaire study (12). For these children, parents completed an extensive sleep behavior questionnaire (the Modified Simonds & Parraga Sleep Questionnaire) (20–22). Cognitive impairment was the most prevalent comorbidity (Supplemental Figure 1A). Seven parents (78%) indicated their child was experiencing sleep problems at the time of completing the questionnaires. The most common problem was early waking (5 out of 7; 71%), defined as waking up before 5 am (Figure 1A). The second most frequent parental complaint was night waking (4 out of 7; 57%) (Figure 1A). Of note, the 2 individuals who had no sleep problems at the time of completing the questionnaire had sleep disturbances in the past, both specifying suffering from night wakings and difficulties falling asleep, and 1 additionally reported early waking and feeling sleepy during the day. We followed the International Classification of Sleep Disorders 3 (ICSD-3) criteria, which define a sleep disorder as complaints occurring at least 3 times per week (23), regardless of whether the caretakers thought their children currently had sleep problems. Parent reports indicated that 56% of children experienced night wakings, and 44% experienced early waking at least 3 times per week (Figure 1B). The frequency of nightly awakenings in the whole cohort ranged from once per night to 3 times per night, with about 55% of them waking twice (Figure 1C). When woken during the night, most remained awake for up to 1 hour, while only 2 resettled in a few minutes (Figure 1D).

In addition to the questionnaires, the parents of the 7 individuals who reported sleep disturbances at that time also provided a well-annotated graphical sleep diary (20–22) for 2 consecutive weeks, except 2 individuals who only completed 1 week. Analysis of these diaries corroborated the observations from the questionnaires and confirmed early morning awakenings, as evidenced by increased wake after sleep offset (WASF) in 5 of 7 individuals with FOXP1 syndrome and by a higher frequency of early awakenings in 4 of 7 patients (Figure 1E and Table 2). Additionally, nearly half of the diaries substantiated elevated wake after sleep onset (WASO), indicating nighttime awakenings, along with low sleep efficiency (below 85%) (Figure 1E and Table 2). One of the most common disruptions was prolonged sleep onset latency (SOL), with 5 individuals showing a median SOL above the reference value of 30 minutes. This finding was unexpected, as it was not a primary complaint. The co-occurrence of increased SOL, WASO, and early morning awakenings was notably prevalent in prepubescent participants (8–13 years old). Total sleep time was in the normal range and largely consistent across the cohort, except for the 2 oldest individuals (13 and 16 years old), who showed reduced and increased sleep, respectively.

Our sleep questionnaire also assessed the effects of the child’s disturbed sleep on family members. All parents and siblings reported being affected by the sleep problems of individuals with FOXP1 syndrome. Not surprisingly, about 80% reported experiencing disrupted sleep due to the child’s sleep problems (Figure 1F). Roughly 55%–66% of family members reported being fatigued and/or feeling irritated and powerless (Figure 1F). About one-third also reported reduced concentration, memory problems, and/or feeling depressed (Figure 1F). Finally, about 20% of parents and siblings reported experiencing feelings of aggression and having more arguments with their partner (Figure 1F). Together, these data highlight sleep disturbances, particularly sleep fragmentation, in individuals with FOXP1 syndrome and their deleterious effects on their families.

### Common variation in FOXP1, FOXP2, and FOXP4 is associated with multiple sleep traits in the general population.

**Figure 2:**
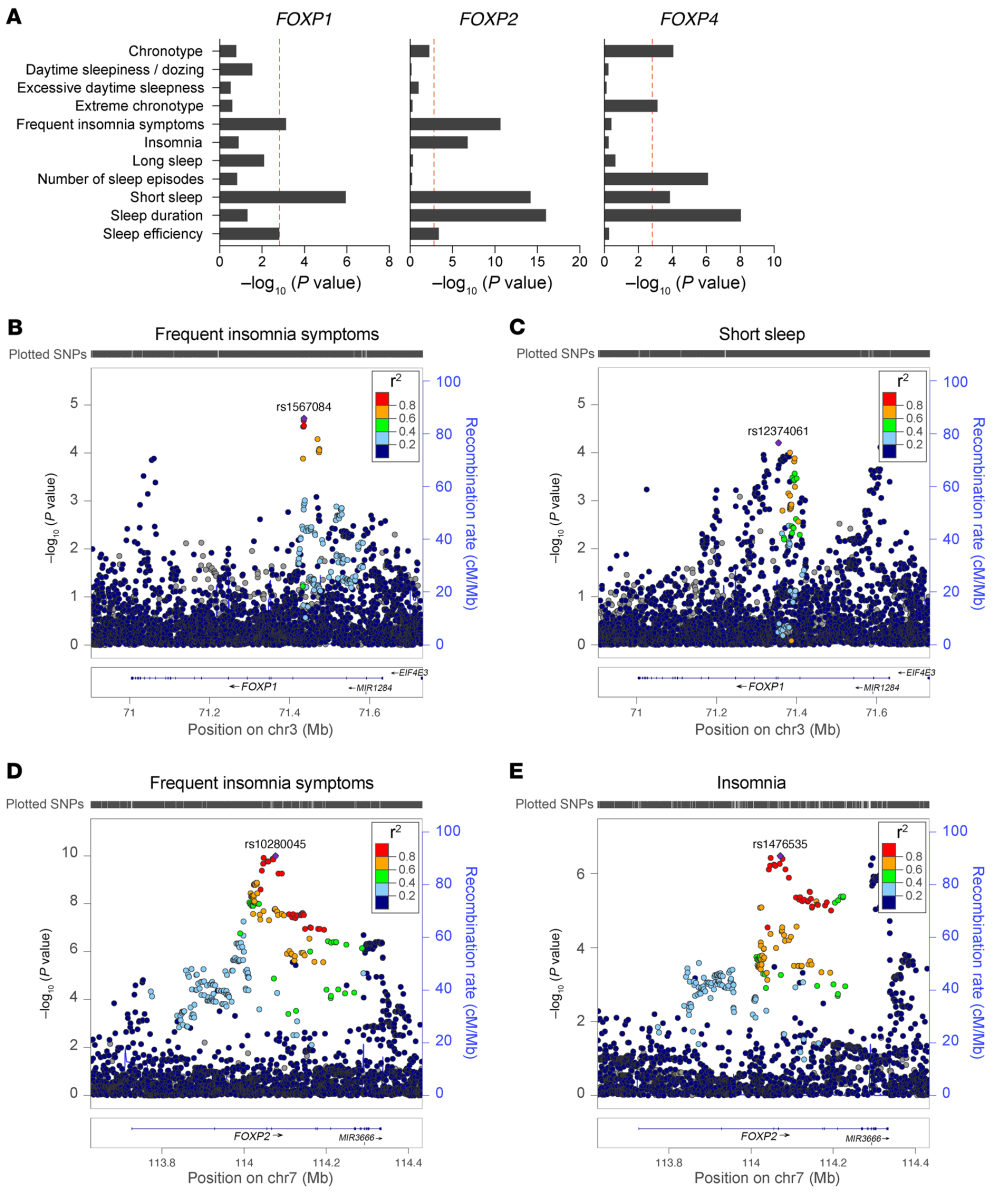
Common variants in *FOXP1*, *FOXP2*, and *FOXP4* are associated with insomnia, frequent insomnia symptoms, and short sleep. (**A**) Gene-based analyses of *FOXP1*, *FOXP2*, and *FOXP4* reveal significant associations with multiple sleep traits. Dashed line indicates significance threshold of *P* < 0.001515 after correction for multiple testing of 11 traits and 3 genes. (**B** and **C**) Regional association plots showing association signals for frequent insomnia symptoms and short sleep at the *FOXP1* locus. (**D** and **E**) Regional association plots showing association signals for frequent insomnia symptoms and insomnia at the *FOXP2* locus. Data are shown as −log_10_(*P* value) for individual SNPs. The color of each marker reflects its linkage disequilibrium (*r*^2^) with the strongest associated SNP indicated as a purple diamond. The recombination rate is indicated in blue. Chr, chromosome.

To obtain further evidence for the importance of FOXP1 in the regulation of sleep, we examined whether common SNPs in the *FOXP1* gene*,* as detected in GWAS, show significant associations with sleep. From an extensive set of GWAS datasets (~4,000 in total) available in the GWAS Atlas (24), we collected data from relevant studies and determined gene-based *P* values for *FOXP1* (NCBI gene ID 27086) as well as for the other members of the FOXP family: *FOXP2* (NCBI gene ID 93986) and *FOXP4* (NCBI gene ID 116113) (Figure 2A and Supplemental Table 2). We were not able to include the X-linked gene *FOXP3* in this analysis due to the insufficient GWAS data for the X chromosome (25). We identified significant associations between common variants in *FOXP1* and the sleep traits frequent insomnia symptoms and short sleep (Figure 2, A–C, and Supplemental Table 3). Similarly, *FOXP2* showed significant gene-wide associations with short sleep and frequent insomnia symptoms as well as with insomnia, sleep efficiency, and sleep duration (Figure 2, A–E, Supplemental Figure 2, A–C, and Supplemental Table 3). *FOXP4* showed associations with short sleep, aligning with *FOXP1* and *FOXP2*, as well as with sleep duration, consistent with *FOXP2*. Furthermore, gene-wide associations of *FOXP4* were significant for chronotype, extreme chronotype, and number of sleep episodes (Figure 2A, Supplemental Figure 3, A–E, and Supplemental Table 3). In summary, gene-wide analyses of common variants of *FOXP1*, *FOXP2*, and *FOXP4* revealed significant associations with several specific sleep traits resembling the sleep disturbances observed in individuals with *FOXP1* gene-disruptive mutations, corroborating a role for the FOXP transcription factor family in sleep regulation.

### Drosophila FoxP mutants recapitulate human sleep maintenance problems.

**Figure 3:**
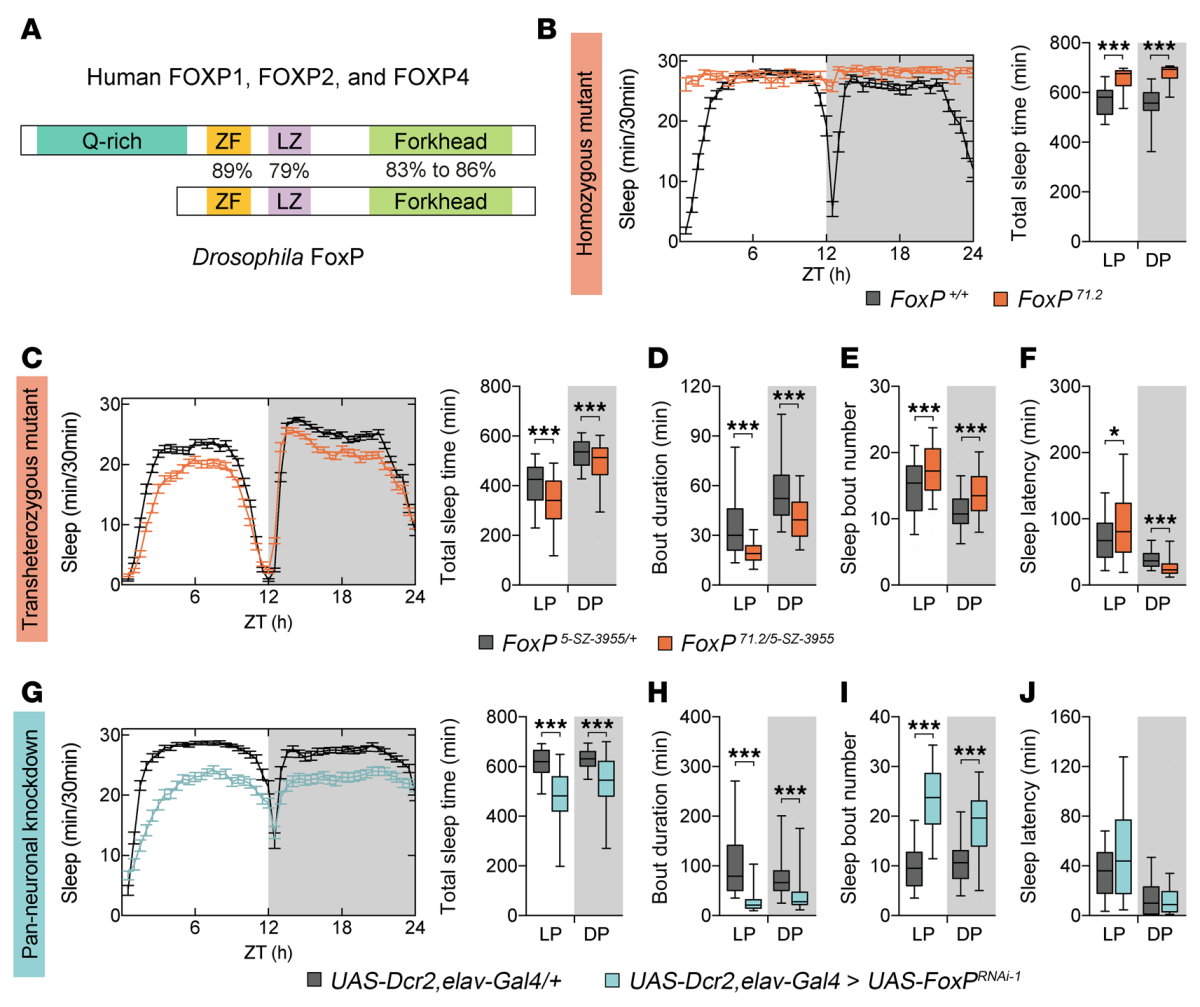
FoxP is required in *Drosophila* neurons to safeguard normal sleep duration and architecture. (**A**) Schematic showing domain conservation between human FOXP1, FOXP2, FOXP4 and their *Drosophila* ortholog, FoxP. Domains include forkhead, zinc finger (ZF), leucine zipper (LZ), and polyglutamine (Q-rich). Adapted from Castells-Nobau et al. (27). (**B**) Representative sleep profile and total sleep time of *FoxP* homozygous mutants (*FoxP^71.2^*, *n* = 14) compared with isogenic controls (*n* = 27). Homozygous *FoxP* mutants show severe locomotor impairments, with high immobility and lack of arousal at dusk/dawn, limiting unbiased sleep assessment. (**C** and **G**) Sleep profiles and their quantification. (**D**, **E**, **H**, and **I**) Average duration (**D** and **H**) and number of sleep bouts (**E** and **I**) in the light (LP, ZT0–12) and dark periods (DP, ZT12–24). *FoxP^71.2/5-SZ-3955^* transheterozygous mutant males (*n* = 117) show reduced total sleep (*P* < 0.0001) (**C**) with shorter sleep bouts (*P* < 0.0001) (**D**), while the number of sleep bouts increased (*P* < 0.0001) (**E**) both during day and night compared with heterozygous *Fox^5-SZ-3955/+^* flies (*n* = 116). (**F** and **J**) Latency to first sleep episode after lights-on and lights-off. (**F**) *FoxP^71.2/5-SZ-3955^* mutants show longer latency to sleep onset after lights-on (*P* = 0.012) and shorter latency after lights-off (*P* < 0.0001). (**G**–**J**) Pan-neuronal *FoxP*-knockdown males (*UAS-Dcr2,elav-Gal4 > UAS-FoxP^RNAi-1^*, *n* = 66) show reduced sleep duration during both the day and night (*P* < 0.0001) compared with isogenic controls (*UAS-Dcr2,elav-Gal4/+*, *n* = 58). Moreover, they exhibit sleep fragmentation, with shorter but more frequent sleep bouts (*P* < 0.0001). SOL is unaffected. Flies were reared at 28°C. Data are presented as box-and-whisker plots showing the 25th to 75th percentiles, with the median indicated; whiskers represent the 5th and 95th percentiles. Statistical analysis was performed using 2-tailed unpaired *t* tests or Mann-Whitney tests, with Bonferroni correction for multiple comparisons. Significance: *P < 0.05 and ***P < 0.001.

To investigate how the *FOXP* gene family regulates sleep, we used the fruit fly *Drosophila melanogaster* as a model. While vertebrates possess 4 *FOXP* paralogs (*FOXP1–4*) derived from gene duplication events, invertebrates like *Drosophila* have a single ortholog, *FoxP* (26). At the protein level, *Drosophila* FoxP is highly conserved with human FOXP1, FOXP2, and FOXP4, particularly in domains required for dimerization and DNA binding: the zinc finger, leucine zipper, and forkhead domains (Figure 3A) (27). A notable difference is the absence of the N-terminal polyglutamine region found in mammalian FOXP proteins, which may influence transcriptional regulation (28). Despite this, *Drosophila* FoxP produces 3 isoforms with distinct forkhead domains that, like their human counterparts, can form homo- and heterodimers to regulate transcription (15, 27). Functionally, *Drosophila* FoxP is essential for neurodevelopment and behaviors relevant to FOXP-associated disorders, including learning, memory, and social behavior (27). These molecular and functional parallels support that *Drosophila* FoxP is the ortholog of FOXP1, FOXP2, and FOXP4.

To address a potential role in sleep regulation of *FoxP*, we first attempted to assess sleep in the *FoxP* null mutants previously generated and characterized (27). Homozygous *FoxP^71.2^* mutants exhibited poor mobility (Figure 3B) consistent with previous findings of substantial locomotor deficits (27), making reliable sleep assessment in *FoxP* null conditions unfeasible. Therefore, we next determined sleep in a less severe yet stringent allele combination. We combined the *FoxP^71.2^* null allele with the hypomorphic allele *FoxP^5-SZ-3955^*. Male *FoxP^71.2/5-SZ-3955^* compound heterozygous mutants did not show motor impairment and exhibited reduced total sleep duration during both the light (20% decrease) and dark (10% decrease) periods when compared with heterozygous *FoxP^5-SZ-3955/+^* controls (Figure 3C). Furthermore, *FoxP^71.2/5-SZ-3955^* flies were unable to maintain sleep for as long as heterozygous mutant flies, which was evidenced by a reduction in sleep bout duration (45% reduction in the day and 30% at night; Figure 3D). This decrease was accompanied by an increase in the number of sleep bouts (Figure 3E), suggesting that *FoxP^71.2/5-SZ-3955^* mutants attempt to compensate for reduced sleep by initiating more bouts. These shorter sleep bouts occurring with a higher number of sleep episodes constitute sleep fragmentation, a hallmark of human sleep maintenance insomnia. The same sleep defects also occurred in compound heterozygous *FoxP^71.2/5-SZ-3955^* female mutants (Supplemental Figure 4, A–C), indicating the function of FoxP in sleep fragmentation is not sex specific. Sleep latency at lights-off, however, was reduced in males and unchanged in females (Figure 3F and Supplemental Figure 4D), contrasting with the increased SOL observed in individuals with FOXP1 syndrome. Overall, *FoxP* loss in *Drosophila* recapitulates the sleep maintenance insomnia observed in FOXP1 syndrome and reflects *FOXP1/2/4*-associated sleep traits in the general population.

### FoxP is required in neurons for sleep integrity.

We sought to collect independent evidence for the role of FoxP in sleep regulation and to identify the cellular substrates through which it acts. Taking advantage of the UAS-Gal4 system and a highly effective and previously validated RNAi line (27, 29, 30), we first induced ubiquitous, constitutive *FoxP* knockdown using *actin-Gal4*. This manipulation recapitulated decreased total sleep time, during both the light and dark periods (Supplemental Figure 5A). Furthermore, ubiquitous *FoxP* knockdown reproduced the sleep fragmentation present in *FoxP^71.2/5-SZ-3955^* mutants (Supplemental Figure 5, B and C). The decreased sleep time and fragmentation were more severe than in compound heterozygous hypomorphic *FoxP* mutants, with sleep bout duration showing a 60% reduction during the day and a 65% decrease during the night. We used an independent, previously validated, weaker RNAi construct (27) to confirm these findings. *FoxP* knockdown with this alternative RNAi faithfully reproduced the daytime and nighttime sleep fragmentation; however, total sleep duration was unaltered (Supplemental Figure 5, D–F).

In the CNS of the fly, FoxP has been shown to be expressed in neurons (27, 31). Therefore, we investigated whether loss of FoxP in neurons would reproduce the sleep disturbances present in *FoxP* mutants. Inducing *FoxP* knockdown with the pan-neuronal driver *elav-Gal4* led to a severe decrease in total sleep time (about a 15%–20% reduction compared with isogenic controls; Figure 3G). Moreover, it reproduced the marked sleep fragmentation seen in the *FoxP^71.2/5-SZ-3955^* mutants and ubiquitous knockdown flies (Figure 3, H–J). In contrast, knocking down *FoxP* in glial cells did not affect sleep duration or structure (Supplemental Figure 5, G–I), supporting the hypothesis that the observed sleep phenotypes originate in neurons rather than glia. Collectively, we conclude that sleep disturbances in *FoxP* mutants map to FoxP function in neurons.

### FoxP is required during development for adult sleep.

**Figure 4:**
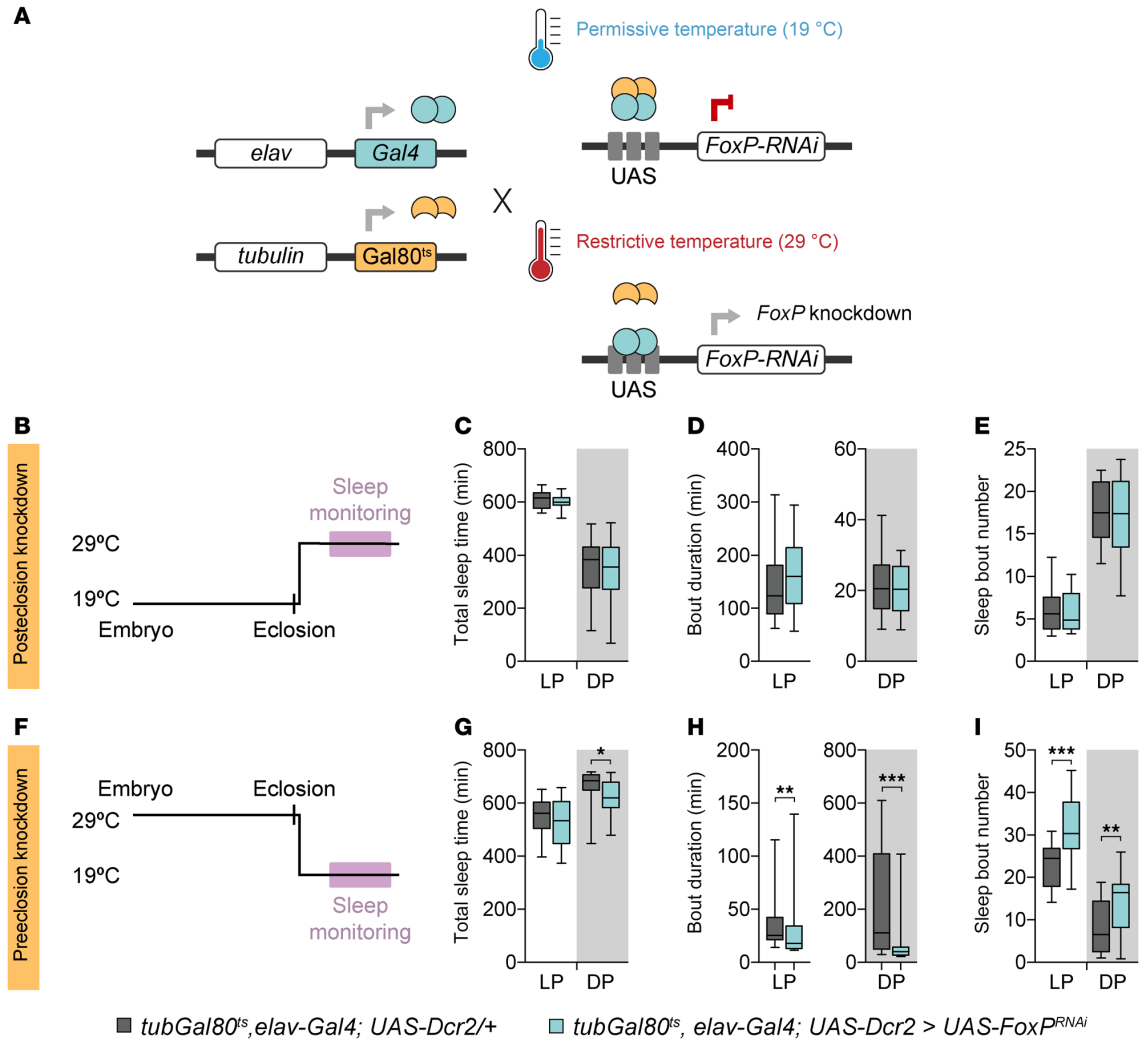
FoxP is required during development for adult sleep. (**A**) Schematic representation of the TARGET system. (**B**) Temperature shifts used to induce posteclosion *FoxP* knockdown, restricting it to adulthood. (**C**–**E**) Representative data for total sleep time (**C**), average sleep bout duration (**D**), and number of sleep bouts (**E**) during the light period (LP, ZT0–12) and dark period (DP, ZT12–24) in flies with pan-neuronal *FoxP* knockdown exclusively during posteclosion stages (*tub-Gal80^ts^, elav-Gal4; UAS-Dcr2 > UAS-FoxP^RNAi-1^*, *n* = 16) compared with isogenic controls (*tub-Gal80^ts^, elav-Gal4; UAS-Dcr2/+*, *n* = 16). Posteclosion *FoxP* knockdown does not affect sleep. (**F**) Temperature shifts used to induce *FoxP* knockdown throughout developmental stages prior to eclosion only. (**G**–**I**) Representative data for total sleep time (**G**), average sleep bout duration (**H**), and number of sleep bouts (**I**) during the light period (ZT0–12) and dark period (ZT12–24) in flies with pan-neuronal *FoxP* knockdown during pre-eclosion stages (*tub-Gal80^ts^, elav-Gal4; UAS-Dcr2 > UAS-FoxP^RNAi-1^*, *n* = 26) compared with isogenic controls (*tub-Gal80^ts^, elav-Gal4; UAS-Dcr2/+*, *n* = 29). *FoxP* knockdown during developmental stages prior to eclosion is necessary and sufficient to cause decreased and fragmented sleep. Data are presented as box-and-whisker plots showing the 25th to 75th percentiles, with the median indicated; whiskers represent the 5th and 95th percentiles. Statistical analysis was performed using 2-tailed unpaired *t* tests or Mann-Whitney tests, with Bonferroni correction for multiple comparisons. Significance: *P < 0.05, **P < 0.01, and ***P < 0.001.

To investigate whether FoxP is required in neurons for sleep integrity early in development or regulates sleep acutely in adulthood, we took advantage of the temporal and regional gene expression targeting (TARGET) system (32) to restrict *FoxP* knockdown temporally (Figure 4A). Flies carrying the *elav-Gal4* driver and the ubiquitous Gal4 repressor *tub-Gal80^ts^* kept at a restrictive temperature (19°C, no *FoxP* knockdown) throughout their lifespan showed comparable sleep duration and architecture to genetic background controls (Supplemental Figure 6, A–D). In contrast, when kept continuously at the permissive temperature (29°C, *FoxP* knockdown), flies showed decreased and fragmented sleep during both the day and the night (Supplemental Figure 6, E–H). Restricting pan-neuronal *FoxP* knockdown to only adulthood (shifting to 29°C immediately after eclosion) did not affect sleep quantity or architecture (Figure 4, B–E). In contrast, *FoxP* knockdown throughout developmental stages prior to eclosion only (29°C pre-eclosion) resulted in decreased and fragmented sleep in the adult, mirroring *FoxP* knockdown throughout the lifespan (Figure 4, F–I). Together, these experiments reveal that FoxP is required during developmental stages in neurons for normal adult sleep.

### FoxP is required for circadian rhythmicity.

**Figure 5:**
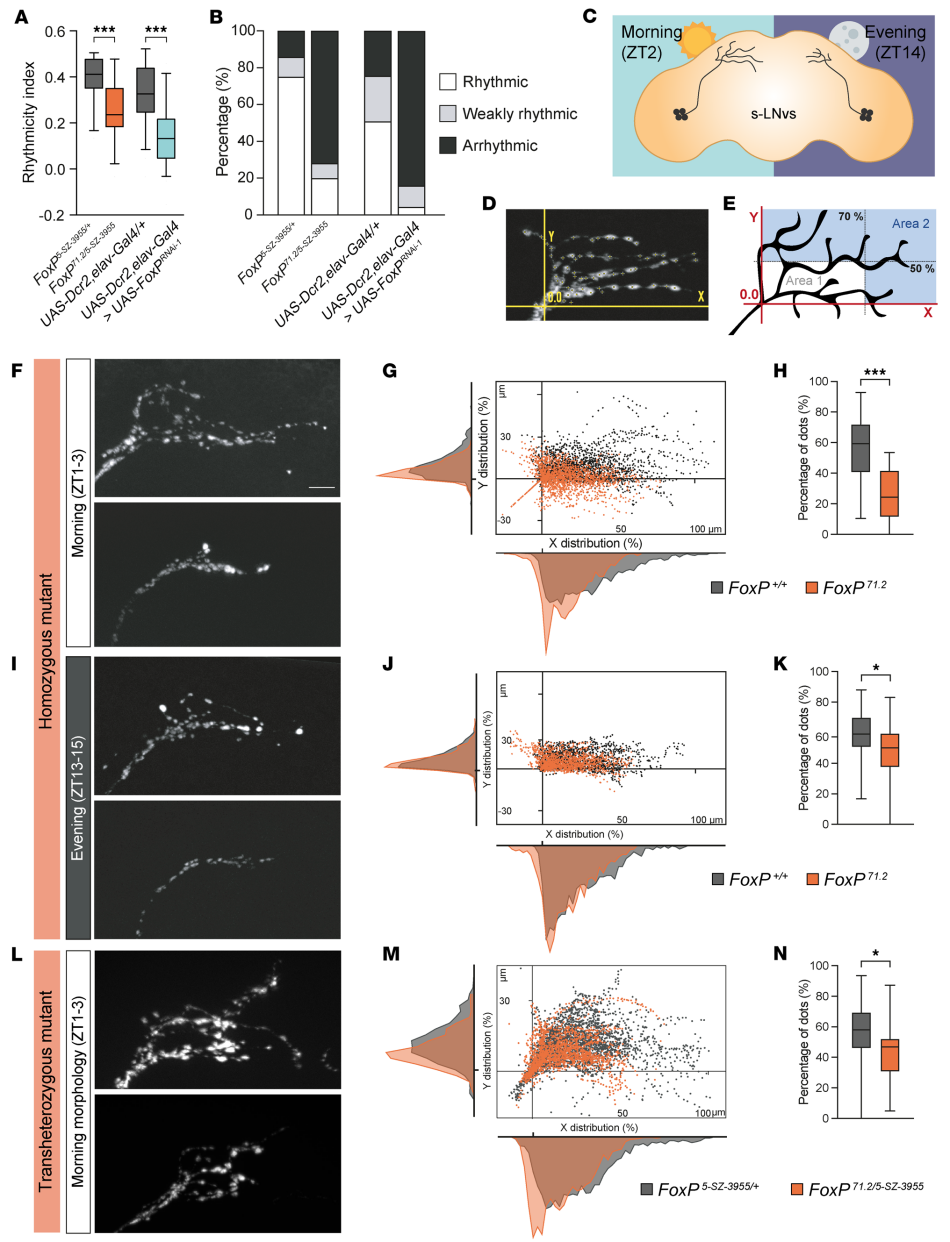
FoxP loss leads to circadian rhythmicity defects and abolishes PDF-secreting s-LNv neuron plasticity. (**A** and **B**) Rhythmicity index (**A**) and proportion of rhythmic, weakly rhythmic, and arrhythmic flies (**B**) in *FoxP^71.2/5-SZ-3955^* compound heterozygous mutant males (*n* = 28) compared with heterozygous hypomorphic *Fox^5-SZ-3955/+^* flies (*n* = 25) and pan-neuronal *FoxP*-knockdown male flies (*UAS-Dcr2,elav-Gal4 > UAS-FoxP^RNAi-1^*, *n* = 69) compared with isogenic controls (*UAS-Dcr2,elav-Gal4/+*, *n* = 61). *FoxP* transheterozygous mutants and *FoxP* pan-neuronal knockdown leads to a lower rhythmicity index and increased arrhythmicity. (**C**) Schematic representation of the presynaptic terminals of s-LNv neurons in the morning (ZT2) and at night (ZT14). During early morning (ZT2), s-LNv projections are highly branched with high levels of PDF immunoreactivity, whereas during the night (ZT14), the projections are decreased and show lower PDF levels (80). (**D** and **E**) Example of synaptic terminal (**D**) and schematic illustration (**E**) of the image segmentation process employed for the quantitative analysis of s-LNv branching morphology. (**F**, **I**, and **L**) Representative images of PDF foci in s-LNv axonal terminals. Scale bar: 10 μm. (**G**, **J**, and **M**) Distribution of Cartesian (*x*, *y*) coordinates for each PDF-immunoreactive maximum. The distributions display the percentage of PDF-immunoreactive maxima along both the *x* and *y* dimensions. (**H**, **K**, and **N**) Percentage of PDF foci within area 2. (**F**–**H**) *FoxP* homozygous mutants (*FoxP^71.2^*, *n* = 30) compared with isogenic controls (*n* = 28) at ZT1–3 (morning). (**I**–**K**) *FoxP* homozygous mutants (*FoxP^71.2^*, *n* = 27) compared with isogenic controls (in gray, *n* = 25) at ZT13–15 (evening). (**L**–**N**) *FoxP^71.2/5-SZ-3955^* compound heterozygous mutants (*n* = 27) compared with heterozygous hypomorphic *Fox^5-SZ-3955/+^* flies (*n* = 25) at ZT1–3 (morning). Data are presented as box-and-whisker plots showing the 25th to 75th percentiles, with the median indicated; whiskers represent the 5th and 95th percentiles. Statistical analysis was performed using a 2-tailed unpaired *t* test. Significance: *P < 0.05 and ***P < 0.001.

Having established a role for FoxP in sleep, we next asked whether FoxP is required for sleep homeostasis by examining whether it affects sleep rebound in response to sleep deprivation. Overnight mechanical sleep deprivation led to strong daytime sleep rebound in *FoxP^71.2/5-SZ-3955^* mutants and their background controls (Supplemental Figure 7, A and B), suggesting that the sleep homeostat remains intact and functions independently of FoxP. Next, we investigated whether FoxP regulates circadian rhythms by assessing rest/activity rhythms in constant darkness. *FoxP* hemizygous mutants showed a decreased rhythmicity index in free-running conditions (Figure 5A). Moreover, only 20% of *FoxP* mutants remained rhythmic compared with 80% of their isogenic controls (Figure 5B). Pan-neuronal *FoxP* knockdown also led to a reduction in rhythm strength in constant darkness (Figure 5, A and B). Compound heterozygous mutant and *FoxP* pan-neuronal knockdown females likewise showed impaired rhythmicity at constant darkness (Supplemental Figure 8A). Thus, FoxP loss disrupts circadian rhythmicity in free-running conditions.

### FoxP affects the plasticity of PDF-secreting neurons in a non-cell-autonomous manner.

Locomotor activity rhythms in *Drosophila* are driven by clock neurons in the brain, whereby the ventrolateral neurons (LNvs) function as central pacemakers that synchronize downstream neuronal oscillators via the rhythmic secretion of the neuropeptide pigment dispersing factor (PDF) (33, 34). The small LNvs (s-LNvs) change electrophysiological properties and presynaptic morphologies between day and night. During the early daytime (Zeitgeber time 1–3 [ZT1–3], peaking 2 hours after lights-on), they exhibit extensive branching of their presynaptic terminals and a higher level of PDF neuropeptide. However, during the early night (ZT14, 2 hours after lights-off), s-LNvs undergo retraction to adopt a less complex morphology with lower levels of PDF (Figure 5B) (35, 36). This plasticity is crucial for downstream connectivity and proper regulation of circadian output behaviors such as sleep, locomotion, and metabolism in a time of day–dependent manner (35, 37).

To investigate whether FoxP regulates s-LNv morphology, we visualized the s-LNvs’ synaptic terminals of *FoxP^71.2^* homozygous null mutants and isogenic controls during the morning (ZT1–3) and evening (ZT13–15) by immunohistochemistry with an α-PDF antibody (Figure 5, C–K). Individual PDF^+^ foci were given a Cartesian (*x*, *y*) coordinate relative to the origin corresponding to the primary s-LNv axonal branching point (*x* = 0, *y* = 0; see Supplemental Methods) (Figure 5, C–E) and were plotted as dispersion graphs. s-LNv axonal terminal of *FoxP^71.2^* homozygous null mutants in the morning (ZT1–3) showed reduced terminal extension compared with isogenic control flies (Figure 5, F–H). We then quantified the percentage of PDF foci in area 2 (Figure 5E), comprising the distal axonal terminals, which confirmed a significant impairment in the distal branching extension of s-LNvs, both in the morning (Figure 5, F–H) and in the evening, albeit to a lesser extent (Figure 5, I–K). Thus, *FoxP* null mutant s-LNv axonal terminals show impaired structural plasticity (Figure 5, F–K, and Supplemental Figure 5, B–E). Moreover, we observed a decrease in s-LNv axonal terminal morning extension and complexity in *FoxP^71.2/5-SZ-3955^* compound heterozygous mutants (Figure 5, L–N). Together, our findings show that loss of FoxP disrupts the plasticity of central pacemaker neurons.

To investigate whether FoxP regulates sleep cell-autonomously in LNvs, we induced *FoxP* knockdown specifically in these neurons using the *Pdf-Gal4* driver. However, LNv-specific *FoxP* knockdown had no effect on sleep duration or its architecture (Supplemental Figure 9, A–C). This result aligns with the lack of *FoxP* expression in PDF neurons during adulthood (Supplemental Figure 9D). We also tested whether FoxP might operate in a subgroup of dorsal clock neurons (DN1), which are contacted by the axonal terminals of the s-LNvs, hypothesizing that they may be involved in the functional defects. We found that *FoxP* knockdown in DN1 neurons led to a significant decrease in daytime sleep (Supplemental Figure 9E) along with fragmented sleep (Supplemental Figure 9, F and G), recapitulating the daytime sleep complaints of the FoxP models.

### FoxP is required in cholinergic, glutamatergic, and peptidergic neurons for sleep.

**Figure 6:**
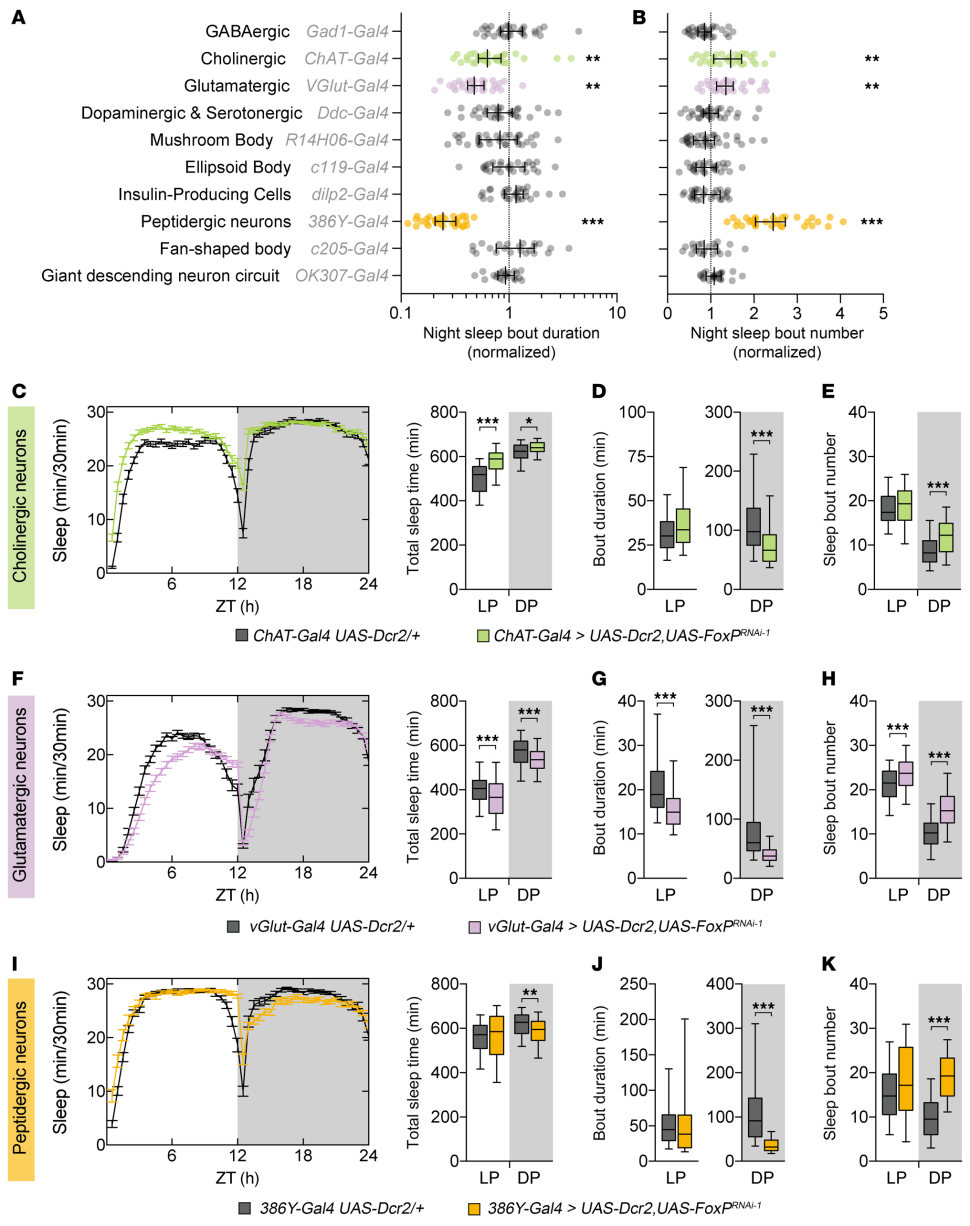
FoxP is required in cholinergic, glutamatergic, and peptidergic neurons for nighttime sleep integrity and duration. (**A**) Effect of *FoxP* knockdown (*UAS-FoxP^RNAi-1^;UAS-Dcr2*) on the average sleep bout duration (**B**) and number of sleep bouts in the different neuronal subtypes. Data were normalized to their respective background controls (*Gal4,UAS-Dcr2/+*). 26–32 flies per genotype. Data are represented as the median ± 95% CI, with individual values depicted. Two-tailed unpaired *t* test or Mann-Whitney *U* test, with Bonferroni correction for multiple testing. (**C**, **F**, and **I**) Sleep profiles and their quantification. (**D**, **E**, **G**, **H**, **J**, and **K**) Average duration (**D**, **G**, and **J**) and number of sleep bouts (**E**, **H**, and **K**) in the light (LP, ZT0–12) and dark periods (DP, ZT12–24). (**C**–**E**) *FoxP* knockdown in cholinergic neurons (*ChAT-Gal4 > UAS-Dcr2,UAS*-*FoxP^RNAi-1^*, *n* = 64) decreases sleep during both day (*P* < 0.0001) and night (*P* = 0.012) compared with controls (*ChAT-Gal4 UAS-Dcr2/+*, *n* = 63). Knockdown flies exhibit shorter but more frequent sleep bouts during the dark period (*P* < 0.0001). (**F**–**H**) *FoxP* knockdown in glutamatergic neurons (*vGlut-Gal4 > UAS-Dcr2,UAS*-*FoxP^RNAi-1^*, *n* = 95) significantly decreases sleep duration during the day (*P* = 0.006) and the night (*P* = 0.00012) compared with controls (*vGlut-Gal4 UAS-Dcr2/+*, *n* = 96). Knockdown flies show shorter but more frequent sleep bouts (*P* < 0.0001, LP and DP). (**I**–**K**) *FoxP* knockdown in neuropeptidergic neurons (*386Y-Gal4 > UAS-Dcr2,UAS*-*FoxP^RNAi-1^*, *n* = 84) leads to a significant decrease in sleep duration at night (*P* = 0.0018) compared with controls (*386Y-Gal4 UAS-Dcr2/+*, *n* = 92). *FoxP*-knockdown flies show shorter (*P* < 0.0001) and an increased number of sleep bouts (*P* < 0.0001). Data are presented as box-and-whisker plots showing the 25th to 75th percentiles, with the median indicated; whiskers represent the 5th and 95th percentiles. Statistical analysis was performed using 2-tailed unpaired *t* test or Mann-Whitney *U* test, with Bonferroni correction for multiple testing. Significance: *P < 0.05, **P < 0.01, and ***P < 0.001.

Although flies sleep during both the day and night, consolidated sleep (longer sleep episodes) preferentially occurs at night and is thought to reflect a crucial deep sleep state, which has been linked to cognitive functioning (38–40). Disruption of consolidated night sleep episodes before reaching deeper stages impairs learning even when total sleep duration remains unaffected (38); in this sense, night sleep fragmentation in *Drosophila* is of greater relevance to the sleep problems experienced by individuals with FOXP1 syndrome. Therefore, we further screened the impact of *FoxP* knockdown in various neurotransmitter systems and brain regions that express FoxP or play a role in sleep regulation (27, 41). *FoxP* knockdown using GABAergic, dopaminergic, and serotonergic drivers and drivers for the mushroom body, the ellipsoid body, the fan-shaped body, the insulin-producing cells, or the giant descending neuron circuit had no effect on the duration or number of nighttime sleep episodes (Figure 6, A and B). However, knockdown in cholinergic, glutamatergic, and peptidergic neurons significantly reduced sleep bout duration while increasing the number of bouts (Figure 6, A and B). Further analysis revealed that in addition to the nighttime sleep fragmentation, *FoxP* knockdown in cholinergic neurons led to increased sleep during the day (Figure 6, C–E). In contrast, *FoxP* knockdown restricted to glutamatergic neurons significantly decreased and fragmented both daytime and nighttime sleep (Figure 6, F–H). Restricting *FoxP* knockdown to peptidergic neurons using the *386Y-Gal4* driver resulted in severe sleep fragmentation. In this case, *FoxP* knockdown exclusively disrupted nighttime sleep (Figure 6, I–K). In conclusion, FoxP is required in glutamatergic neurons for both daytime and nighttime sleep and in cholinergic and especially peptidergic neurons for nighttime sleep.

### FoxP loss leads to the downregulation of genes involved in neuropeptidergic signaling.

**Figure 7:**
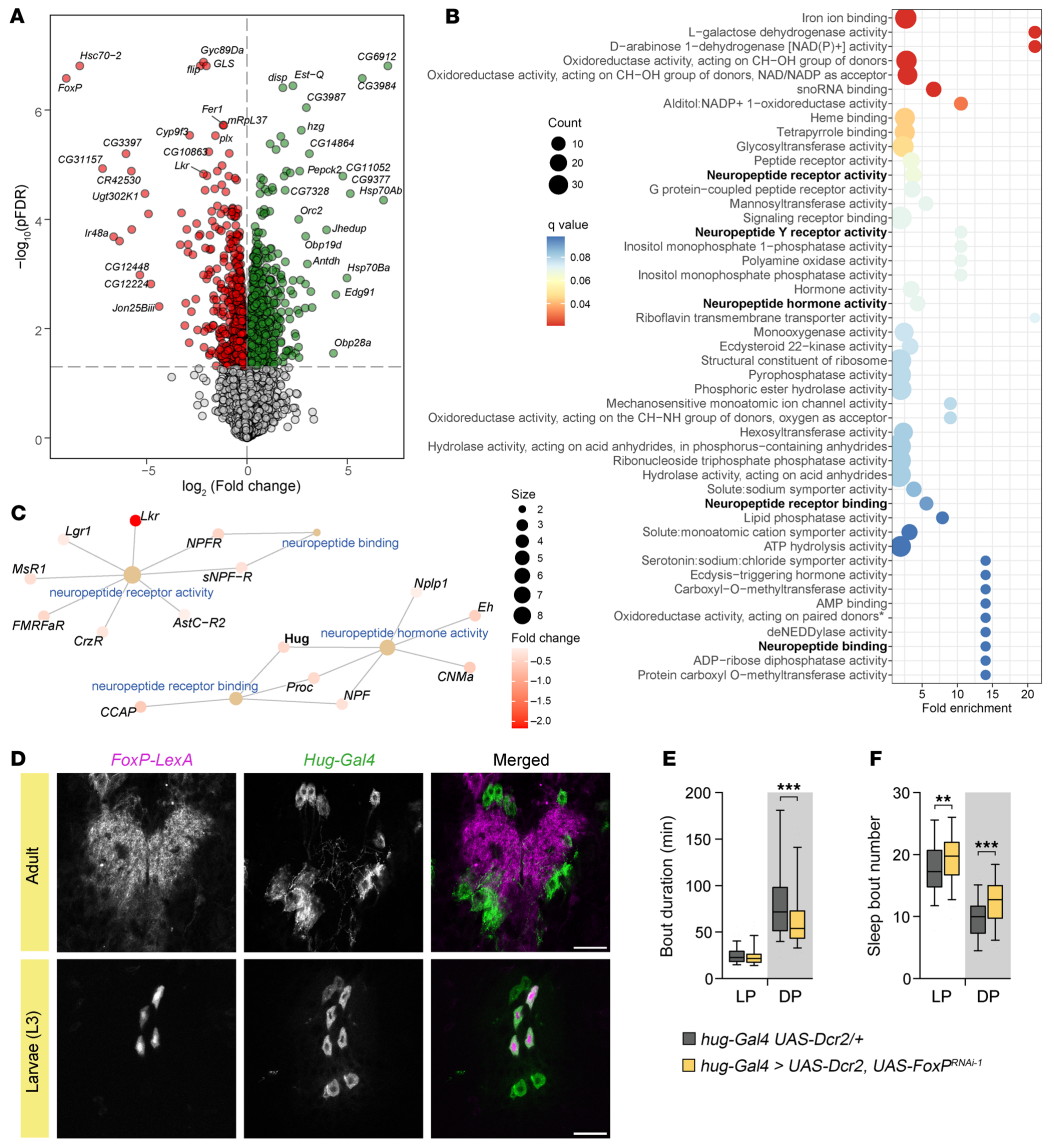
FoxP regulates genes involved in neuropeptide signaling and controls sleep integrity in peptidergic hugin^+^ neurons. (**A**) Volcano plot of differentially expressed genes in the brains of *FoxP^71.2^* homozygous mutants identified using robust linear regression models (*t* statistic based on M estimation with Huber weighting solved using iteratively reweighted least squares). The log_2_ of the fold change and the log_10_ *P* values adjusted for multiple testing (pFDR) are plotted for each gene. Differentially expressed genes (pFDR < 0.05) are colored in red and green, indicating down- and upregulation, respectively. (**B**) Dot plot of GO molecular function enrichment analysis from genes significantly downregulated in the brains of *FoxP^71.2^* homozygous mutants. Dots are colored according to their *q* value. (**C**) Gene concept network illustrating significant genes associated with key molecular functions: neuropeptide receptor activity, neuropeptide binding, neuropeptide hormone activity, and neuropeptide receptor binding. (**D**) Colocalization of FoxP^+^ and hugin^+^ neurons; *FoxP-LexA* and *Hug-Gal4* were combined with *LexOp-mCD8-RFP* and *UAS-mCD8-GFP*, respectively, to identify overlapping fluorescent signal at adult and wandering L3 larval stages. FoxP is partially expressed in hugin^+^ neurons in L3 larvae brains, but not in adults. Scale bars: 25 μm. (**E** and **F**) Average duration (**E**) and number of sleep bouts (**F**) during the light (LP, ZT0–12) and dark periods (DP, ZT12–24). *FoxP* knockdown in hugin^+^ neurons (*hug-Gal4 > UAS-Dcr2, UAS*-*FoxP^RNAi-1^*, *n* = 95) leads to shorter but more frequent sleep bouts exclusively during the night (*P* = 0.0004 and *P* < 0.0001) compared with controls (*hug-Gal4 UAS-Dcr2/+*, *n* = 96). Data are presented as box-and-whisker plots showing the 25th to 75th percentiles, with the median indicated; whiskers represent the 5th and 95th percentiles. Statistical analysis was performed using 2-tailed unpaired *t* test or Mann-Whitney *U* test with Bonferroni’s correction for multiple testing. Significance: **P < 0.01 and ***P < 0.001.

FoxP is a transcription factor and, as such, functions by regulating downstream gene expression programs. To gain insight into the specific FoxP target genes that mediate sleep, we performed transcriptomics analysis (RNA-seq) of *FoxP* null mutant and isogenic control whole brains. To evaluate consistency between the biological replicates, we first performed principal component analysis, which revealed a clear separation between the 2 genotypes (Supplemental Figure 10A). We identified 1,575 significantly differentially expressed genes (809 up- and 766 downregulated genes) and confirmed that, as expected, the gene with the largest negative fold change in expression is *FoxP* itself (Figure 7A and Supplemental Table 4). Remarkably, Gene Ontology (GO) molecular function overrepresentation analysis of significantly downregulated genes revealed that FoxP regulates targets involved in neuropeptidergic function such as neuropeptide receptor activity and binding, neuropeptide binding, neuropeptide hormone activity, and neuropeptide receptor binding, among others (Figure 7, B and C, and Supplemental Table 5). Similarly, we identified pathways related to neuropeptide signaling with overrepresentation analysis of the downregulated targets at the GO biological process level (Supplemental Figure 10, B and C, and Supplemental Table 6). Given that peptidergic neurons underlie the night sleep fragmentation (Figure 6, I–K), the finding that FoxP regulates genes involved in neuropeptidergic function is of particular interest.

### FoxP promotes nighttime sleep integrity via its function in hugin^+^ neurons.

Motivated by the role of FoxP in peptidergic neurons — where it regulates sleep and transcriptionally regulates neuropeptide activity — we investigated whether FoxP exerts its effects through one of its downstream targets. Since we recapitulated the sleep maintenance defect observed in *FoxP* mutants by knocking down *FoxP* specifically in peptidergic neurons using the *386-GAL4* driver (Figure 6), we examined the neuronal populations targeted by this construct in more detail. The *386Y-Gal4* driver follows the pattern of the *amontillado* gene, which encodes a prohormone convertase essential for processing neuropeptide precursors into bioactive hormones. These include adipokinetic hormone, FMRFamide-like peptides 2–8, CAPA peptides (CAPA-periviscerokinin-1/2 and CAPA-pyrokinin), corazonin, myosuppressin, Hugin (Hug), and short neuropeptide F-1 and -2 (42, 43). We examined the expression of genes encoding these neuropeptides in the targets identified by RNA-seq in *FoxP* mutant brains. Surprisingly, only one of these peptidergic hormones, Hugin, was significantly downregulated in *FoxP^71.2^* homozygous null mutants (Figure 7C and Supplemental Figure 10C), suggesting a specific link between FoxP and Hugin-expressing neurons in sleep regulation.

Hugin is a prepropeptide that produces 2 neuropeptides, Hugin-γ and pyrokinin-2, the latter homologous to mammalian neuromedin U (44). To investigate whether FoxP is expressed in hugin^+^ neurons, we used genetically encoded fluorescent proteins to colabel FoxP and hugin^+^ neurons. We used the driver *hug-Gal4* to express *UAS-GFP* in about 20 hugin^+^ neurons in the subesophageal zone and labeled FoxP-expressing cells by expressing *LexOp-mCD8-RFP* using a previously validated FoxP driver, *FoxP-LexA* (45). We found that FoxP was undetectable in hugin^+^ neurons during adulthood (Figure 7D). However, motivated by the developmental origin of FoxP-dependent sleep fragmentation, we also investigated whether FoxP is expressed in hugin^+^ neurons during earlier stages of development. We found that at the wandering L3 larval stage, FoxP is expressed in a subset of hugin^+^ neurons (Figure 7D). Next, we investigated whether FoxP may regulate sleep integrity in these peptidergic neurons. Indeed, we found that *FoxP* knockdown in hugin^+^ neurons led to sleep fragmentation at nighttime, as evidenced by shorter but more frequent sleep bouts (Figure 7, E and F). Together, we conclude that FoxP is required during development in a subset of hugin^+^ neurons for proper adult sleep architecture.

Finally, to explore whether FOXP1/2/4 may regulate neuromedin U, we analyzed recent high-resolution single-cell RNA-seq datasets of the human cortex at prenatal (46) and postnatal stages (47). In the developing cortex, *NMU* — the gene encoding neuromedin U in humans — is expressed mainly in the medial ganglionic eminence (dividing and intermediate cells), in dividing intermediate progenitor cells, and in dividing radial glia (Supplemental Figure 11A). *FOXP1* and *FOXP2* are expressed in these clusters, while *FOXP4* only shows sparse expression. In the adult cortex, *NMU* is highly localized to somatostatin and parvalbumin neuronal clusters (Supplemental Figure 11B). *FOXP1* and *FOXP4* are broadly expressed in these cells, whereas *FOXP2* expression is more limited and at lower levels. Together, these data support a role for FOXP1 in *NMU*-expressing neurons during both development and adulthood in the human cortex.

## Discussion

Sleep disturbances represent a major burden for individuals with NDDs, as they are highly pervasive and have a severe negative impact on their well-being and quality of life and that of their families. Therefore, it is crucial to gain a deeper understanding of their etiology to facilitate the development of therapeutic approaches. In the present study, we show that individuals with FOXP1 syndrome suffer from night awakenings and early waking, and to a lesser extent sleep onset problems and daytime sleepiness. We demonstrate that common variation in the FOXP family is associated with related sleep traits in the general population, indicating a conserved role for this transcription factor family in sleep regulation. Moreover, we establish that these sleep problems are recapitulated in a preclinical *Drosophila* model of the syndrome. In addition, *FoxP* mutants show severe circadian defects. In *Drosophila*, *FoxP* is required during development in neurons, particularly peptidergic neurons, to regulate sleep integrity later in life, likely due to the role of FoxP in the transcriptional regulation of peptidergic signaling.

By extracting sleep data from 2 large ASD studies alongside detailed sleep phenotyping via questionnaires and sleep diaries in a smaller independent cohort, we found that individuals with disruptive *FOXP1* mutations suffer from night awakenings and early waking (before 5 am), indicative of sleep maintenance insomnia. These disturbances occurred at rates comparable to those in individuals with idiopathic or other forms of monogenic ASD, substantially exceeding both the previously reported 5% prevalence in *FOXP1* syndrome (9) and estimates for neurotypical populations (2, 11). Moreover, investigating prior GWAS data, we report that common variation (indexed by SNPs) within *FOXP1*, *FOXP2*, and *FOXP4* is associated with multiple sleep traits, including frequent insomnia symptoms and short sleep. Interestingly, these traits resemble the sleep disturbances we observed in FOXP1 syndrome and the sleep problems reported in FOXP2 syndrome (13). While we do not claim these prevalence estimates are definitive, we believe they offer a meaningful overview of the types of sleep disturbances observed in FOXP1 syndrome and highlight the role of the FOXP gene family in sleep regulation.

Additionally, to our knowledge, we provide the first functional evidence from an animal model showing that loss of the *Drosophila* ortholog *FoxP* recapitulates sleep disturbances linked to *FOXP* family genes. Specifically, *FoxP* mutants exhibited reduced and fragmented daytime and nighttime sleep, characterized by shorter but more frequent sleep bouts. Overall, *FoxP* loss in *Drosophila* models sleep maintenance insomnia observed in FOXP1 syndrome and mirrors *FOXP1/2/4*-associated sleep traits in the general population. Together, these data also exclude that sleep disturbances in FOXP1 syndrome are merely secondary to other developmental impairments, arguing that the FOXP family are physiological regulators of sleep. One notable difference between our clinical and preclinical findings was the number of sleep episodes, which remained within the typical range in the *FOXP1* syndrome cohort but was consistently altered in our fly models. This discrepancy might reflect ethological differences between species, but it could also indicate that sleep fragmentation in FOXP1 syndrome is underestimated. Studies in neurotypical children show that caregiver-reported diaries and questionnaires often miss subtle night awakenings, capturing only major disruptions. This leads to overestimation of total sleep time, sleep efficiency, and time in bed compared with actigraphy and polysomnography (48, 49). It is therefore plausible that both WASO and the number of awakenings are underestimated, while sleep duration is overestimated in this FOXP1 syndrome cohort.

In contrast, sleep scheduling parameters such as onset and offset times show greater concordance between sleep diaries and actigraphy (50). We observed a widespread increase in SOL documented by the diaries in the FOXP1 syndrome cohort. This result was unexpected, as in both the initial study (12) and our sleep questionnaire, this remained unreported. Additionally, no consistent changes in sleep latency were observed in our *Drosophila* models. Future studies using quantitative tools like actigraphy or polysomnography are needed to reveal the full extent of these disturbances. Together, our findings provide the first evidence implicating the *FOXP* family in sleep regulation across monogenic cohorts, the general population, and an animal model.

We observed that FoxP loss in flies disrupts rest/activity rhythms under free-running conditions, as both *FoxP* mutants and *FoxP* pan-neuronal knockdown animals exhibited impaired rhythmicity in constant darkness. These rhythms are driven by clock neurons, with the LNvs functioning as central pacemakers that synchronize downstream neuronal oscillators through rhythmic PDF neuropeptide secretion (33, 34). The s-LNvs display day–night changes in electrophysiological properties and presynaptic morphology, characterized by extensive branching and elevated PDF levels in the morning (ZT2), which retract and exhibit reduced PDF levels during the early night (ZT14) (35, 36). We found that the axonal terminals of s-LNvs in *FoxP* mutants (both homozygous null and compound heterozygous) exhibited reduced distal branching extension during both the morning and evening, with a more pronounced reduction in the morning. This indicates that FoxP loss, in a non-cell-autonomous manner, not only impairs s-LNv axonal terminal extension but also prevents these neurons from adopting their elaborated morning morphology, thereby abolishing circadian plasticity. Further studies are needed to elucidate the precise mechanisms by which FoxP loss disrupt s-LNv circadian plasticity.

We identified the s-LNv circuit output cluster, the dorsal clock neuron cluster DN1, as a contributor to the daytime sleep disturbances observed in *FoxP* mutants. While the role of clock neurons in promoting arousal is well established (51, 52), their involvement in sleep regulation through circadian mechanisms is less understood. DN1 neurons modulate activity in response to environmental cues such as light and temperature (53). They have been shown to promote both daytime siesta and nighttime sleep; optogenetic activation of DN1 neurons increases midday siesta sleep, while their inhibition reduces it (54). Additionally, blocking DN1 neurotransmitter release decreases both siesta and nighttime sleep (54). It thus appears conceivable that FoxP loss in DN1 neurons leads to diminished activity of the s-LNv–DN1 circuit.

To better understand the cellular and temporal dynamics underlying FoxP regulation of sleep, we restricted FoxP knockdown in a spatial and temporal manner. This allowed us to determine that FoxP is required in neurons during developmental stages to regulate sleep later during adulthood. Furthermore, our results indicate that sleep disturbances in FoxP models arise from the function of this gene in multiple neuronal populations, especially of peptidergic neurons, with more modest contributions from cholinergic and glutamatergic neurons. Interestingly, recent studies in mice have shown that FOXP1 plays a critical role in strengthening and maturation of glutamatergic inputs to the striatum (55), suggesting a conserved role for the FOXP1/FoxP family in regulating glutamatergic transmission across species. We were particularly interested in mapping and understanding the alterations in nighttime sleep architecture, given its conserved critical role in supporting higher cognitive functions in both humans and flies (38, 56). The strongest disruption of nighttime sleep architecture in the shape of sleep fragmentation was observed when FoxP loss was restricted to neuropeptidergic neurons with the *386Y-Gal4* driver. Moreover, FoxP loss led to a striking downregulation of genes involved in various components of neuropeptidergic function, emphasizing its importance in maintaining proper neuropeptidergic signaling and sleep regulation.

We further identified that FoxP is required in neurons expressing and secreting the neuropeptide hugin and that its loss in these neurons leads to sleep fragmentation. Hugin^+^ neurons are located in the subesophageal zone, a sensorimotor control center in flies, and are known to regulate locomotion and feeding during both larval and adult stages (57–59). *Hugin* knockdown in these neurons in adulthood was shown to weaken rest/activity rhythms (60). Similarly, acutely silencing or ablating hugin^+^ neurons reduced the amplitude of rest/activity rhythms (60). In contrast, thermogenetic activation of the hugin^+^ neuronal cluster in adulthood increased locomotion and decreased feeding (57). It was reported that neither acute activation nor silencing of hugin^+^ neurons affects sleep duration (61). Interestingly, hugin^+^ neurons play a critical role in linking the circadian clock to rest/activity rhythms. They integrate clock input originating from PDF-secreting central pacemaker s-LNv neurons via DN1 neurons and diuretic hormone 44–secreting neurons to regulate motor outputs through projections to the ventral nerve cord (60). Although *hugin* mRNA levels do not cycle, the rhythms of neuropeptide release in the ventral nerve cord from hugin^+^ neurons are regulated by the circadian clock (60). While this suggests that under physiological conditions hugin levels may not be rate-limiting for release and rest/activity rhythms, reduced levels of hugin in FoxP^+^ neurons may become limiting. Hugin^+^ neurons also integrate circadian and sleep signals to modulate circadian circuitry to ultimately regulate sleep timing as they also receive input from the dorsal fan-shaped body (dFB), a core component of the fly sleep homeostat (61). Upon sleep deprivation, the activity of hugin^+^ neurons decreases to putatively suppress circadian-driven activity during the sleep recovery phase. Conversely, ablation of hugin^+^ neurons enhances the dFB-driven sleep increase (61). Notably, hugin^+^ neurons project to and feed back onto PDF-secreting s-LNv clock neurons, enabling these neurons to respond in a hugin-dependent manner to sleep loss (61). Whether any of these processes are affected by loss of FoxP and how it might relate to the general FoxP phenotypic spectrum remain to be determined.

The involvement of neuropeptides in FOXP1 syndrome and the sleep pathophysiology associated with FOXP family members remain unknown. However, neuropeptides are known key regulators of the sleep-wake cycle in mammals (62). Interestingly, the proposed vertebrate homolog of hugin, neuromedin U (44), has been implicated in sleep regulation across multiple animal models. In rats, neuromedin U injection disrupts sleep architecture (63), and its overexpression in zebrafish increases arousal and reduces sleep (64). Moreover, we demonstrate that *FOXP1* is expressed in the same cortical neurons as *NMU*, the gene encoding neuromedin U. This raises the possibility that members of the FOXP family may regulate sleep in humans through the (transcriptional) regulation of this and/or other neuropeptides affecting multiple neuroendocrine systems. For instance, in the murine brain stem, all neuropeptide S-expressing neurons express high levels of *Foxp2* (65, 66). Neuropeptide S has been shown to promote arousal and increase wakefulness in mice (67). Additionally, mutations in the *neuropeptide S receptor 1* gene have been associated with reduced sleep duration and delayed bedtime in humans (68–70), with the former phenotype replicated in a mouse model carrying the same mutation (70).

Neuropeptidergic function is not only critical to regulating sleep but also for various other essential physiological processes and behaviors. In this context, the hypothalamus plays a central role in homeostatic regulation through neuroendocrine signaling (71). Recent studies have revealed that *Foxp1* expression defines a cluster of neurons secreting the neuropeptide oxytocin in the murine hypothalamus (72). This link might be important for understanding behaviors seen in FOXP1 syndrome, including those related to autism (73). Beyond the CNS, in the mouse airway epithelium, Foxp1 and Foxp4 jointly restrict neuropeptide Y (NPY) expression, and the loss of both genes leads to increased NPY levels (74). Interestingly, we found that the *Drosophila* ortholog of NPY — neuropeptide F (NPF) — and its receptors (75) are dysregulated in *FoxP* mutants (Figure 7C). As both NPY and NPF are prominently expressed in the CNS (76, 77), it is plausible that FoxP/FoxP1 regulate these same genetic targets in the brain across species, suggesting that FOXP1 regulates neuropeptidergic function in humans. Our findings provide evidence supporting a conserved and broader role for the FOXP family in regulating neuropeptidergic signaling. Furthermore, to our knowledge, we offer the first evidence that sleep disturbances in FOXP1 and FOXP2 syndromes may, at least in part, arise from impaired neuropeptidergic function and suggest that the same mechanisms help regulate sleep traits in the general population. Although neuropeptide-based therapies are in early stages, they may represent an inroad to ameliorate sleep and other behavioral problems in FOXP family–related disorders (78, 79).

## Methods

### Sex as a biological variable.

Sex as a biological variable was considered by assessing sleep in individuals with FOXP1 syndrome regardless of gender and by including both male and female animals in functional sleep characterization to investigate potential sex-specific differences. Findings were similar for both sexes.

### Human and Drosophila studies.

A detailed description of sleep assessment procedures in the FOXP1 syndrome cohorts, as well as the *Drosophila* stocks and experimental assays used, is provided in Supplemental Methods.

### Statistics.

Statistical analyses were performed in R (version 4.3.1) or in GraphPad Prism version 10 for Windows (GraphPad Software). Fisher’s exact tests were used to assess differences between individuals with sleep disturbances with disruptive *FOXP1* variants and either an idiopathic ASD group or a group with LGDMs in other genes, with correction for multiple comparisons applied using FDR.

For *Drosophila* sleep experiments, to assess significance between 2 genotypes, we used 2-tailed unpaired *t* tests for data following a Gaussian distribution or Mann-Whitney tests for nonparametric data. Bonferroni’s post hoc correction was further applied to these analyses to account for the number of tests performed per genotype, and only *P* values passing this corrected significance level are indicated in the figures. The rhythmicity index was compared between genotypes using 2-tailed unpaired *t* tests. Sleep rebound ability was assessed for each genotype by comparing total sleep during ZT0–3 under baseline conditions and during the same period after sleep deprivation, using a 2-tailed paired Student’s *t* test. The percentages of immunoreactive PDF points within area 2 across genotypes were compared using a 2-tailed unpaired Student’s *t* test. All data are representative of at least 3 independent experiments (*N* ≥ 3) unless otherwise specified.

For the analysis of FoxP targets identified via RNA-seq, *P* values were adjusted for multiple comparisons using the Benjamini-Hochberg procedure to control the FDR. Pathway enrichment significance was then assessed using a hypergeometric test, with a Storey procedure (*q* values) applied for multiple testing correction. Only *P* values less than 0.05 after correction for multiple testing (where applicable) were considered significant.

### Study approval.

Written informed consent was obtained from all participants, including the 6 individuals with *FOXP1* mutations recruited from the SSC and an ongoing study funded by an NIH grant to Evan E. Eichler (R01MH101221), as well as participants in the idiopathic ASD cohort and the cohort with LGDMs in other NDD genes, both also drawn from the SSC. All procedures were approved by the Institutional Review Board of the University of Washington. Additionally, written consent was obtained from the parents of children with FOXP1 syndrome who had previously reported sleep problems in the Parent-Reported Phenotype of FOXP1 Syndrome study (12) and agreed to participate in this follow-up sleep study. The study protocol was reviewed and approved by the Medical Ethics Committee of Leiden–Den Haag–Delft, the Netherlands (protocol number N21.085).

### Data availability.

Gene expression data are available in the Gene Expression Omnibus with the accession number GSE293784. The values for all data points in the graphs are reported in the Supporting Data Values file.

## Author contributions

MCT, IE, ACN, and AS conceptualized the study. IE, LVVR, SP, and ACN developed the methodology. IE, JMP, and ACN were responsible for software development. MCT, IE, JH, NR, LVVR, CMH, JMP, MK, SK, and ACN performed the experiments and formal analyses. MCT, SK, ACN, and AS wrote the original draft. All authors reviewed and approved the manuscript. MCT, JMP, MK, and ACN created the visualizations and figures. MCT, SEF, MSK, TK, ACN, and AS supervised the project. MCT, SEF, TK, MK, ACN, and AS acquired the funding necessary to conduct the work described in this manuscript.

## Funding support

- Radboudumc personal PhD fellowship and a travelling fellowship by The Company of Biologists (DMMTF1908278) to MCT.
- Radboudumc junior researcher fellowships (2) to SEF and AS, and to TK and AS.
- Netherlands Organisation for Scientific Research grants 09150162010073 (ZonMw Veni to MK), 91718310 (ZonMw Vidi to TK), and 09150181910022 (ZonMw Vici to AS).
- Max Planck Society support to SEF.
- Instituto de Salud Carlos III (ISCIII; Madrid, Spain) funding through the Miguel Servet Program CP24/00033, cofunded by the European Union under the European Social Fund “Investing in your future” to ACN.
- ISCIII grant PI25/00392, cofunded by the European Union, to ACN.

## Supplementary Material

Supplemental data

Supplemental table 3

Supplemental table 4

Supplemental table 5

Supplemental table 6

Supporting data values

## Figures and Tables

**Table 1:**
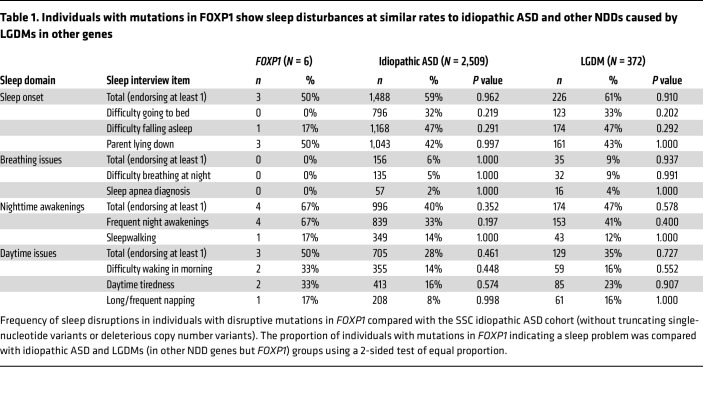
Individuals with mutations in FOXP1 show sleep disturbances at similar rates to idiopathic ASD and other NDDs caused by LGDMs in other genes

**Table 2:**
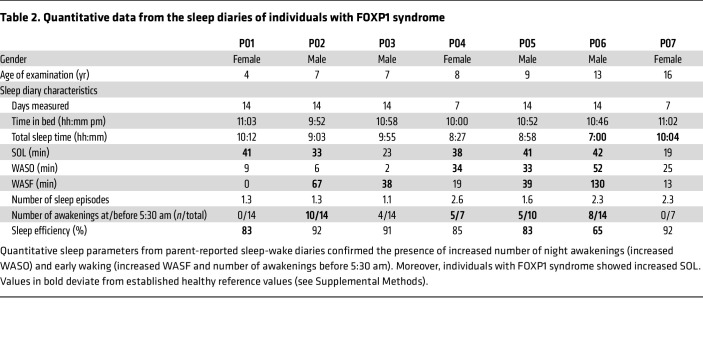
Quantitative data from the sleep diaries of individuals with FOXP1 syndrome

## Version 1 04/01/2026

Electronic publication
