# Supplementary material for: Conserved sleep disturbances in FOXP1 syndrome originate from developmental dysregulation of peptidergic signaling
Source: J Clin Invest. 2026 Apr 1;136(7):e193475. doi: 10.1172/JCI193475 (PMC13038207; doi:10.1172/JCI193475)
Supplement: Supplemental data [file jci-136-193475-s239.pdf]

**Gender**	Male	Male	Female	Female	Female	Female
**Age** (months)	113	138	117	133	117	248
**ASD diagnosis**	Yes	Yes	Yes	No	Yes	Yes
**ID diagnosis**	Yes	No	Yes	Yes	Yes	No
***FOXP1* diagnosis**	p.Ala339Serfs*4	p.Gln60Arg	p.G531_534del	p.Leu414Ter	p.Y470C	p.Ala575CysfsTer9
	c.1014dupA	c.179A>G	c.1590_1601del	c.1240delC	c.1409A>G	c.1722_1722+1insTGCAGCTTTACAG
**Variant classification**	Likely pathogenic	Likely pathogenic	Pathogenic	Likely pathogenic	Likely pathogenic	Pathogenic
**Inheritance**	*de novo*	*de novo*	*de novo*	*de novo*	*de novo*	*de novo*
**Reported sleep problems**						
Sleep onset	Yes	Yes	No	No	No	Yes
Nighttime awakenings	Yes	Yes	Yes	No	No	Yes
Breathing issues	No	No	No	No	No	No
Daytime issues	No	No	Yes	No	Yes	Yes

Supplemental Table 1 – Individuals with mutations in *FOXP1* from the SSC cohort included in our study. Related to Table 1.

Atlas ID	PMID	Year	N	Domain	Trait	Gene-based *p-value FOXP1*	Gene-based *p-value FOXP2*	Gene-based *p-value FOXP4*
4290	30804566	2019	237627	Neurological	Frequent insomnia symptoms	**7.4E-04**	**2.2E-11**	0.395
3786	30804565	2019	386533	Neurological	Insomnia	0.127	**1.6E-07**	0.561
1141	27494321	2016	128266	Psychiatric	Chronotype	0.163	0.0055	**0.0001**
3790	30804565	2019	385333	Psychiatric	Daytime sleepiness / dozing	0.029	0.6810	0.586
1175	27992416	2017	111648	Psychiatric	Excessive daytime sleepiness	0.305	0.0970	0.758
1174	26955885	2016	100420	Psychiatric	Extreme chronotype	0.255	0.5242	**0.0008**
4273	30846698	2019	339926	Psychiatric	Long sleep	0.008	0.4684	0.227
4252	30952852	2019	84810	Psychiatric	Number of sleep episodes	0.149	0.5938	**7.9E-07**
4274	30846698	2019	411934	Psychiatric	Short sleep	**1.1E-06**	**6.5E-15**	**1.4E-04**
4272	30846698	2019	446118	Psychiatric	Sleep duration	0.049	**1.0E-16**	**9.3E-09**
4255	30952852	2019	84810	Psychiatric	Sleep efficiency	0.0015	**4.1E-04**	0.538

Supplemental Table 2 – Common variation in *FOXP1*, *FOXP2*, and *FOXP4* is associated with multiple sleep traits. Gene-based analyses of FOXP1, FOXP2, and FOXP4 reveal significant associations with multiple sleep traits, including frequent insomnia symptoms and short sleep. Bonferroni correction for multiple comparisons was applied to the significance level [for 33 tests (11 traits for 3 genes): $p < 0.001515$]. Values withstanding correction for multiple testing are in bold.

Supplemental Materials and Methods

Sleep assessment of FOXP1 individuals related to a large-scale of autistic children (SCC)

We assessed sleep characteristics in a cohort of individuals with *FOXP1* mutations using structured questionnaires. We included all individuals identified (a total of six) with variants that disrupt *FOXP1* (Supplemental Table 1): one individual was drawn from SSC (1, 2) and the other five from an ongoing study of individuals with pathogenic variants in ASD-associated genes [NIH grant to Evan E. Eichler (R01MH101221)] (3). Pathogenic or likely pathogenic variants in *FOXP1* were confirmed through a review of clinical or research genetic testing results; individuals with other pathogenic variants in NDD- and/or sleep-related genes were excluded from analyses. Given the lack of an independent, age-matched neurotypical control group that had undergone the same in-depth genetic and phenotypic assessments, we established two different comparison group(s) drawn from the SSC: (i) a cohort of individuals with ASD but no known genetic etiology (idiopathic ASD), 2509 probands without pathogenic ASD-associated single-gene variants or deleterious copy number variants as confirmed by whole-exome sequencing, and (ii) a cohort of individuals with LGDM in other NDD genes (2, 4). Detailed information on the idiopathic ASD cohort used can be found in (5). Written consent was obtained from participants, and all procedures were approved by the University of Washington Institutional Review Board.

A comprehensive, structured medical history interview initially designed for the large-scale SSC was completed with primary caregivers of all participants, which included the same questions about history of sleep problems. Caregivers were asked whether their child had ever experienced the sleep problems, including bedtime problems, excessive daytime sleepiness, sleep-disordered breathing, and nighttime awakenings.

Sleep assessment in individuals with FOXP1 syndrome

Parents of children with FOXP1 syndrome who reported sleep problems in the “parent-reported phenotype of FOXP1 syndrome” study (6) were re-contacted and invited to participate in this follow-up sleep study. Patients of all ages carrying a (likely) pathogenic variant in *FOXP1* or a deletion affecting *FOXP1* were eligible for inclusion. Individuals with larger deletions encompassing additional genes beyond *FOXP1* were excluded from the original study and, consequently, from the present follow-up study. The medical ethics committee of Leiden-Den Haag-Delft, the Netherlands, reviewed and approved the study protocol (N21.085). Parents of nine individuals with FOXP1 syndrome agreed to participate and after informed consent were asked to complete the Modified Simonds & Parraga Sleep Questionnaire (7-9) digitally using the survey package from Castor EDC. Additionally, they

were asked to fill a graphical sleep diary for two consecutive weeks (7-9). Of note, three children used melatonin, while four others were on psychoactive medications (pipamperone, guanfacine, methylphenidate and aripiprazole, fluoxetine). One child took medication for an overactive bladder, and another one for allergy.

Sleep parameters were calculated from the sleep diaries using the average of the person's sleep indices and compared to age-appropriate reference values. The following parameters were assessed with a 15-minute bin resolution based on parent-reported sleep-wake calendars: time in bed (TIB), total sleep time (TST), sleep onset latency (SOL), wake after sleep onset (WASO), wake after sleep finish (WASF), and sleep efficiency (SE). SE was calculated as (TST/TIB × 100) and considered deviant if below 85%. SOL was defined as the time between going to bed and sleep onset. WASO represented the total time awake between sleep onset sleep offset. WASF was calculated as the total time awake between sleep offset and the end of TIB. Deviance for the normal range was determined per individual with FOXP1 syndrome by comparing their averages to established reference values for age-matched neurotypical children or adolescents (10, 11). Since no reference values exist for WASF in children—only in adults, where it is typically one minute (12)—we set a threshold of 30 minutes, considering that children, particularly those with disabilities, may require more time before leaving bed. Likewise, no established reference values exist for the number of awakenings before 5:30 a.m. However, based on the ICSD-3 criteria, which define a sleep disorder as complaints occurring at least three times per week (13), we used a threshold of three or more nights per week with early waking.

Gene-based associations of *FOXP1*, *FOXP2*, and *FOXP4*

On 28 June 2021, we derived gene-based *p*-values for *FOXP1* (NCBI Gene ID: 27086), *FOXP2* (NCBI Gene ID: 93986) and *FOXP4* (NCBI Gene ID: 116113) for all available GWAS (~4,000) in the GWAS atlas (14) for sleep-related traits (GWAS $N = 11$). We queried the GWAS results of the subchapters 'Sleep Functions' (Domain: Psychiatric; Chapter: Mental Functions) and 'Sleep Disorders' (Domain: Neurological; Chapter: Diseases of the Nervous System). If duplicated traits were identified, we selected the GWAS that included the largest sample size and the GWAS that used combined data from men and women. In the GWAS atlas, for gene-based analyses, MAGMA v1.06 (15) was used. MAGMA gene analysis was performed using 19,436 protein coding genes obtained from biomaRt (primary ID is Ensembl ID v92 GRCh37) that are assigned to the NCBI Entrez ID. SNPs are assigned to genes with a 1 Kb window on each side. For all gene-based analyses, the default model, SNP-wide (mean), was used.

Drosophila stocks and husbandry

Flies were reared on standard medium containing yeast, cornmeal, agar, and sugar at 25°C, 60% humidity, and entrained in a 12:12 LD cycle unless stated otherwise. The following *Drosophila* stocks were used for this study: w^{+}*;; FoxP*$^{+/+}$ (generated in-house), w^{+}*;; FoxP*$^{71.2}$*/Tm6C,Sb* [generated in-house (16)], w^{1118}*;; FoxP*$^{5\text{-}SZ\text{-}3955}$ (#126252) was obtained from the Kyoto Stock Center (DGRC), background control for Vienna *Drosophila* Resource Center (VDRC) GD library (VDRC #60000), *UAS-FoxP*$^{RNAi\text{-}1}$ (VDRC #15732), *UAS-Dicer2/CyO* in the VDRC GD library background (generated in-house), *UAS-Dicer2/CyO; UAS-FoxP*$^{RNAi\text{-}1}$*/TM6C* (generated in-house), *UAS-FoxP*$^{RNAi\text{-}2}$ (VDRC #15735), *UAS-Dcr2/Y; actin-Gal4/CyO-GFP*, *UAS-Dcr2; elav-Gal4*, *UAS-Dcr2/CyO; repo-Gal4/TM3,Sb*(17), *tubGal80*^{ts}*/CyO* in the VDRC GD library background (generated in-house), *tubGal80*^{ts}*/CyO; UAS-FoxP*$^{RNAi\text{-}1}$*/TM6b,Tb* (generated in-house), *Pdf-Gal4,UAS-Dcr2/CyO* (gift from Sebastian Kadener), *Clk4.5F-Gal4/TM6B,Tb*, *Gad1-Gal4/CyO* [(Bloomington Drosophila Stock Center (BDSC) #51630], *VGlut-Gal4* (BDSC #26160), *ChAT-Gal4/CyO*, *Ddc-Gal4* (BDSC #7010), *R14H06-Gal4* (BDSC #48667), *dilp2-Gal4/CyO*, *c119-Gal4* (BDSC #30824), *c205-Gal4* (BDSC #30826), *OK307-Gal4* (BDSC # 6488), *386Y-Gal4* (gift from Paul Taghert), *Hug-Gal4* (BDSC #58769), and *lexAop-mCD8::RFP,UAS-mCD8::GFP;FoxP-lexA/TM3* (gift from Björn Brembs). Of note, the *FoxP*$^{5\text{-}SZ\text{-}3955}$ allele, a P-element insertion, is reported to affect only isoform 2 of *FoxP* (18).

Sleep monitoring and circadian rhythmicity

Locomotor activity and sleep were recorded with the Trikinetics *Drosophila* Activity Monitor (DAM2) system (Waltham, MA, USA). Briefly, 3- to 5-day-old male flies (unless specified otherwise) were individually placed without CO_2 anesthesia in transparent tubes (65 mm x 5 mm) containing standard food, loaded into the DAM systems, and allowed to acclimate to activity monitors and food for at least 12 hours before starting the experiment. Flies were monitored for four days at 25°C in a 12:12 LD cycle, unless specified otherwise, followed by five days in constant darkness (DD). Motion was detected via the monitors' infrared light beams, and the parameters of activity and sleep (defined in *Drosophila* as five or more minutes of inactivity) (19, 20) parameters were extracted using the publicly available Sleep and Circadian Analysis MATLAB Program (SCAMP) (21) for MATLAB. The sleep data presented are the average for the four days of data acquisition, unless otherwise specified.

Circadian analysis

To determine the rhythmicity of individual flies, we performed an autocorrelation analysis of the data to define their rhythmicity index (RI) (22). Flies were divided into three different

groups according to their RI: (i) Rhythmic (RI > 0.3), when there was the presence of clear peaks during DD; (ii) weakly rhythmic (0.2 < RI < 0.3), when there was the presence of vague peaks during DD; and (iii) arrhythmic (RI < 0.2), when there was a lack of morning/evening peaks during DD.

TARGET system experiments

We used the temperature-dependent TARGET system (23) for temporal mapping experiments. Flies were reared at either 19°C (restrictive temperature) or 29°C (permissive temperature), and after eclosion (day 0) they were either kept at the same temperature or swapped as indicated to confine gene knockdown to pre- or post-eclosion periods. Genetic background controls were reared in parallel with the experimental flies. All experiments were conducted in a 12:12 LD cycle.

Mechanical sleep deprivation

Mechanical sleep deprivation was performed as previously described (17). Experimental flies loaded on DAM2 monitors were placed on a Trikinetics vortexer mounting plate (Waltham, MA, USA), and their activity and sleep were recorded for three consecutive days. On the second day, the monitors were shaken for two seconds randomly within every 20 second window for a total of 12 hours, comprising the dark period (ZT 12-24). The rebound ability of each genotype was assessed by comparing total sleep during ZT 0-3 on the previous day in unperturbed conditions (baseline) and the same period after sleep deprivation (recovery).

S-LNv axon morphology imaging and quantification

Adult brains of 3-to-6-day-old flies were dissected during ZT1-3 (morning) or ZT13-15 (evening) and fixed for 30 minutes in 3.7% paraformaldehyde (PFA) in phosphate-buffered saline (PBS). After fixation, the brains were washed with PBS with 0.3% Triton X-100 (PBS-T) for 30 minutes twice and then incubated in blocking solution containing 5% normal goat serum (NGS) in 0.3% PBS-T for 2 h at room temperature with continuous shaking. The samples were incubated with mouse α-PDF C7 (Developmental Studies Hybridoma Bank; AB_760350) diluted 1:5 in PBS-T with 1% NGS for three days at 4 °C with continuous shaking. Brains were sequentially washed five times for 15 minutes in PBS-T at room temperature and then incubated with the secondary antibody Alexa-568 red α-mouse (#A-11004, ThermoFisher Scientific) diluted 1:500 in PBS-T and 0.5% NGS for two days at room temperature with continuous shaking. Brains were washed five times for 15 minutes in PBS-T at room temperature and were subsequently mounted in ProLong™ Gold Antifade Mountant (ThermoFisher Scientific). Samples were stored at -20°C until further analysis.

To visualize axon projections of s-LNvs, optical sections of whole brains were imaged on a Zeiss Axio Imager Z1 fluorescence microscope. Stacks were taken with a 63X objective (oil immersion), at a distance of 1µm to obtain a reconstruction of the full trajectory of these axons. Images were analyzed using FIJI. PDF-immunoreactive foci from s-LNvs terminals were identified using the "*Find Maxima*" function. Each identified maxima point was assigned X and Y coordinates relative to the origin of a Cartesian coordinate system (X = 0, Y = 0). The origin was manually defined for each individual neuron at the primary branching point of the s-LNvs postsynaptic terminal projections. The unique distribution of PDF-immunoreactive terminals for each genotype was visualized using dot plots to provide an overview of terminal dispersion. The spatial distribution was analyzed with R; the first quadrant of the coordinate system was subdivided into two distinct areas: (i) area 1 (white included PDF-immunoreactive points concentrated near the primary axonal branching point (the defined Cartesian origin), and (ii) area 2 (blue) captured points farther from the origin, providing an indication of the degree of branching (Figure 5E). The threshold to define areas 1 and 2 was determined by the values encompassing 50% of the PDF-immunoreactive foci along the X-axis and 70% along the Y-axis, based on the pooled sum of PDF foci from all terminals analyzed in the respective control genotypes. These thresholds were then applied to all individual neurons in both control and experimental datasets. Boxplots were generated to display the percentages of immunoreactive PDF points within the distal area (area 2) for individual neurons across genotypes.

FoxP co-localization

Brains from L3 larvae and 3-day-old adult flies were dissected in PBS and fixed with 4% PFA for 20 minutes. After fixation, brains were washed 3X with 0.3% Triton X-100 in PBS, and then immediately mounted on glass slides with Vectashield H-1000 and sealed. Stack images were captured on a Zeiss LSM 880 confocal microscope. Max projections were made from the stacks to assess the overlap between FoxP-expressing cells (with the *FoxP-LexA* construct) and either hugin^{+} neurons or PDF-secreting neurons (with the *Hug-Gal4* or the *Pdf-Gal4* drivers, respectively).

RNA-sequencing and data analysis

Twelve brains of freshly eclosed males per sample were dissected in PBS at ZT1-2, transferred to RNAlater solution (Sigma), and immediately frozen in liquid nitrogen. Four biological replicates were performed per genotype. Total RNA was extracted using the Arcturus PicoPure RNA Purification kit (Thermo Fisher Scientific) and the TruSeq RNA Sample Preparation Kit (Illumina) was used to prepare adapter ligated PCR fragments for sequencing. mRNA was purified using polyA selection and was reverse transcribed into a

single-stranded cDNA using random hexamer priming. Second strand synthesis was performed to create double-stranded cDNA. The cDNA was then summited to DNA end repair with adenylation followed by ligation of Illumina sequencing adaptors and size selection for 300 bp. Fragments were amplified linearly with 14 PCR cycles and the sample quality of the fragments was validated using the 2100 Agilent Bioanalyzer. Single indexed samples were multiplexed and sequenced on an Illumina HiSeq 2500 sequencing system (Illumina) in single-end mode with a read length of 36 bp, according to standard protocols of the manufacturer. 30-68 million reads were obtained per sample. Sequenced reads were aligned with Burrows-Wheeler algorithm (24) to the *Drosophila* reference genome with gene annotations extracted from the file Drosophila_melanogaster.BDGP5.77.gtf, available at http://www.ensembl.org (BDGP.5, http://www.fruitfly.org/) allowing one mismatch. Read counts were generated per gene with Rsubread v1.16.1 (25). Only tags that were uniquely aligned to the genome were considered for further analysis. A total of 15–56 million reads with high quality exon alignment were obtained for each sample, showing a total alignment efficiency between 60-74%.

Differential expression gene analyses were performed on gene counts using the ‘limma’ R package(26). Low expressed genes were filtered, and only genes with more than ten reads in at least three samples were selected for further analysis. RNA-seq data were then normalized for RNA composition using the trimmed mean of M-value as implemented in the edgeR package (27). Normalized counts were then converted to log2 count per million (logCPM) using the ‘voom’ function. A robust linear regression model was then fitted to the data using the ‘lmFit’ function with the option method of ‘robust’, to limit the influence of outlying samples. Finally, an empirical Bayes method was applied to borrow information between genes with the ‘eBayes’ function. The functional roles of differentially expressed genes were characterized using overrepresentation analyses based on GO using ClusterProfiler.

NMU and *FOXP1/2/4* gene family expression in human cortex

The t-stochastic neighbor embedding (tSNE) projections along with the cluster annotations were extracted from the original datasets for both the developing (28) and the adult human cortex (29). The single-cell RNA-seq counts per million (CPM) for both datasets were processed into UMI counts using quantile normalization (30). SCTransform (31) was applied on the post quantile normalized count matrix. The plots for expression are the log-normalized expression corrected through SCTransform. Data was visualized with the UCSC Cell Browser (32).

Supplemental References

1. Fischbach GD, and Lord C. The Simons Simplex Collection: a resource for identification of autism genetic risk factors. *Neuron.* 2010;68(2):192-5.
2. Sanders SJ, He X, Willsey AJ, Ercan-Sencicek AG, Samocha KE, Cicek AE, et al. Insights into Autism Spectrum Disorder Genomic Architecture and Biology from 71 Risk Loci. *Neuron.* 2015;87(6):1215-33.
3. Beighley JS, Hudac CM, Arnett AB, Peterson JL, Gerdts J, Wallace AS, et al. Clinical Phenotypes of Carriers of Mutations in CHD8 or Its Conserved Target Genes. *Biol Psychiatry.* 2020;87(2):123-31.
4. Iossifov I, O'Roak BJ, Sanders SJ, Ronemus M, Krumm N, Levy D, et al. The contribution of de novo coding mutations to autism spectrum disorder. *Nature.* 2014;515(7526):216-21.
5. Earl RK, Ward T, Gerdts J, Eichler EE, Bernier RA, and Hudac CM. Sleep Problems in Children with ASD and Gene Disrupting Mutations. *J Genet Psychol.* 2021;182(5):317-34.
6. Koene S, Ropers FG, Wieland J, Rybak T, Wildschut F, Berghuis D, et al. Clinical phenotype of FOXP1 syndrome: parent-reported medical signs and symptoms in 40 individuals. *J Med Genet.* 2024;61(4):399-404.
7. Simonds JF, and Parraga H. Prevalence of sleep disorders and sleep behaviors in children and adolescents. *J Am Acad Child Psychiatry.* 1982;21(4):383-8.
8. Simonds JF, and Parraga H. Sleep behaviors and disorders in children and adolescents evaluated at psychiatric clinics. *J Dev Behav Pediatr.* 1984;5(1):6-10.
9. Wiggs L, and Stores G. Behavioural treatment for sleep problems in children with severe learning disabilities and challenging daytime behaviour: effect on sleep patterns of mother and child. *J Sleep Res.* 1998;7(2):119-26.
10. Paruthi S, Brooks LJ, D'Ambrosio C, Hall WA, Kotagal S, Lloyd RM, et al. Recommended Amount of Sleep for Pediatric Populations: A Consensus Statement of the American Academy of Sleep Medicine. *J Clin Sleep Med.* 2016;12(6):785-6.
11. Mollayeva T, Thurairajah P, Burton K, Mollayeva S, Shapiro CM, and Colantonio A. The Pittsburgh sleep quality index as a screening tool for sleep dysfunction in clinical and non-clinical samples: A systematic review and meta-analysis. *Sleep Med Rev.* 2016;25:52-73.
12. Keller M, Roth WT, and Petrowski K. Supplementing sleep actigraphy with button pressing while awake. *PLoS One.* 2020;15(6):e0234060.
13. Medicine AAoS. International classification of sleep disorders—third edition (ICSD-3). *AASM Resour Libr.* 2014;281:2313.

14. Watanabe K, Stringer S, Frei O, Umićević Mirkov M, de Leeuw C, Polderman TJC, et al. A global overview of pleiotropy and genetic architecture in complex traits. *Nat Genet.* 2019;51(9):1339-48.
15. de Leeuw CA, Mooij JM, Heskes T, and Posthuma D. MAGMA: generalized gene-set analysis of GWAS data. *PLoS Comput Biol.* 2015;11(4):e1004219.
16. Castells-Nobau A, Eidhof I, Fenckova M, Brenman-Suttner DB, Scheffer-de Gooyert JM, Christine S, et al. Conserved regulation of neurodevelopmental processes and behavior by FoxP in Drosophila. *PLoS One.* 2019;14(2):e0211652.
17. Coll-Tané M, Gong NN, Belfer SJ, van Renssen LV, Kurtz-Nelson EC, Szuperak M, et al. The CHD8/CHD7/Kismet family links blood-brain barrier glia and serotonin to ASD-associated sleep defects. *Sci Adv.* 2021;7(23).
18. Mendoza E, Colomb J, Rybak J, Pflüger HJ, Zars T, Scharff C, and Brembs B. Drosophila FoxP mutants are deficient in operant self-learning. *PLoS One.* 2014;9(6):e100648.
19. Shaw PJ, Cirelli C, Greenspan RJ, and Tononi G. Correlates of sleep and waking in Drosophila melanogaster. *Science.* 2000;287(5459):1834-7.
20. Hendricks JC, Finn SM, Panckeri KA, Chavkin J, Williams JA, Sehgal A, and Pack AI. Rest in Drosophila is a sleep-like state. *Neuron.* 2000;25(1):129-38.
21. Donelson NC, Kim EZ, Slawson JB, Vecsey CG, Huber R, and Griffith LC. High-resolution positional tracking for long-term analysis of Drosophila sleep and locomotion using the "tracker" program. *PLoS One.* 2012;7(5):e37250.
22. Levine JD, Funes P, Dowse HB, and Hall JC. Signal analysis of behavioral and molecular cycles. *BMC neuroscience.* 2002;3:1.
23. McGuire SE, Roman G, and Davis RL. Gene expression systems in Drosophila: a synthesis of time and space. *Trends Genet.* 2004;20(8):384-91.
24. Li H, and Durbin R. Fast and accurate long-read alignment with Burrows-Wheeler transform. *Bioinformatics.* 2010;26(5):589-95.
25. Liao Y, Smyth GK, and Shi W. The R package Rsubread is easier, faster, cheaper and better for alignment and quantification of RNA sequencing reads. *Nucleic Acids Res.* 2019;47(8):e47.
26. Ritchie ME, Phipson B, Wu D, Hu Y, Law CW, Shi W, and Smyth GK. limma powers differential expression analyses for RNA-sequencing and microarray studies. *Nucleic Acids Res.* 2015;43(7):e47.
27. Robinson MD, McCarthy DJ, and Smyth GK. edgeR: a Bioconductor package for differential expression analysis of digital gene expression data. *Bioinformatics.* 2010;26(1):139-40.

28. Nowakowski TJ, Bhaduri A, Pollen AA, Alvarado B, Mostajo-Radji MA, Di Lullo E, et al. Spatiotemporal gene expression trajectories reveal developmental hierarchies of the human cortex. *Science (New York, NY).* 2017;358(6368):1318-23.
29. Hodge RD, Bakken TE, Miller JA, Smith KA, Barkan ER, Graybuck LT, et al. Conserved cell types with divergent features in human versus mouse cortex. *Nature.* 2019;573(7772):61-8.
30. Townes FW, and Irizarry RA. Quantile normalization of single-cell RNA-seq read counts without unique molecular identifiers. *bioRxiv.* 2019:817031.
31. Hafemeister C, and Satija R. Normalization and variance stabilization of single-cell RNA-seq data using regularized negative binomial regression. *Genome Biol.* 2019;20(1):296.
32. Speir ML, Bhaduri A, Markov NS, Moreno P, Nowakowski TJ, Papatheodorou I, et al. UCSC Cell Browser: visualize your single-cell data. *Bioinformatics.* 2021;37(23):4578-80.

Supplemental Figures

A

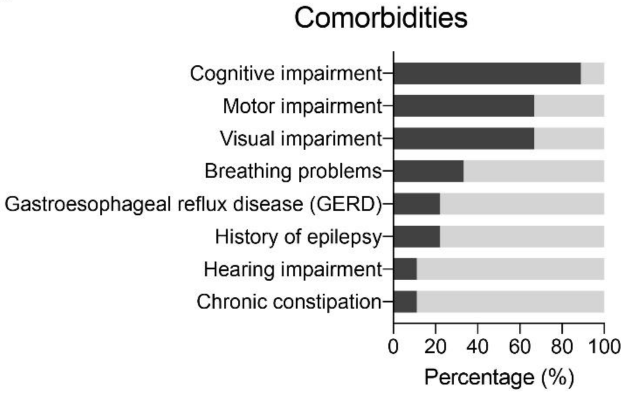

Supplemental Figure 1 – Parent-reported comorbidities present in our FOXP1 syndrome cohort. (**A**) Prevalence of the comorbidities reported in the nine individuals that participated in our sleep questionnaire.

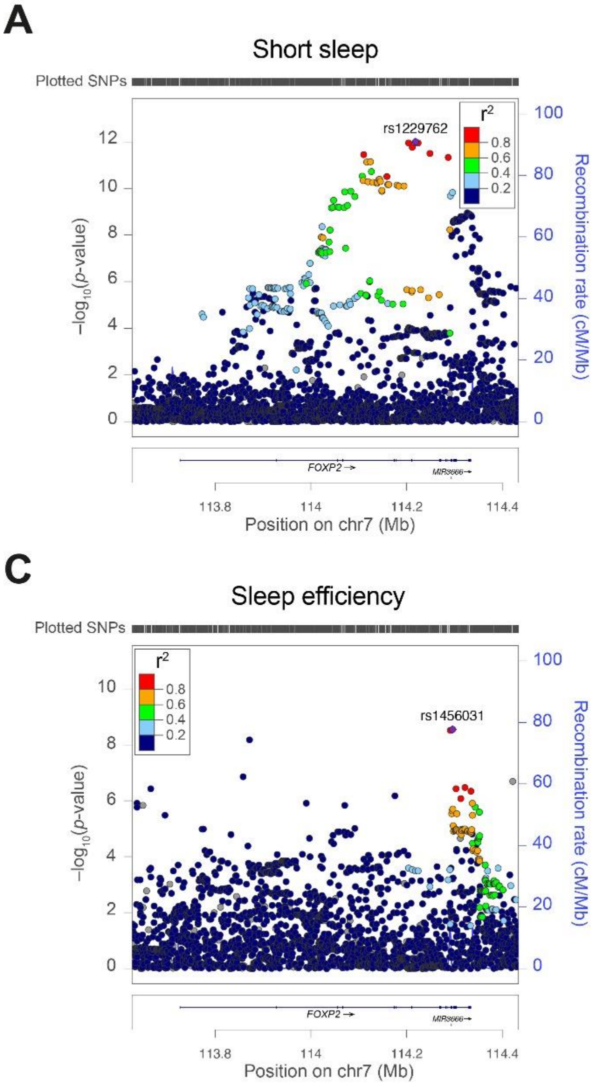

Supplemental Figure 2 – SNPs in *FOXP2* are associated with short sleep, sleep duration, and sleep efficiency. Regional association plots showing association signals for (**A**) short sleep, (**B**) sleep duration, and (**C**) sleep efficiency at the *FOXP2* locus. Data are shown as $-\log_{10}$(*p*-value) for individual SNPs. The color of each marker reflects its linkage disequilibrium (r^2) with the strongest associated SNP indicated as a purple diamond. The recombination rate is indicated in blue. Chr, chromosome; cM, centimorgan; Mb, megabase.

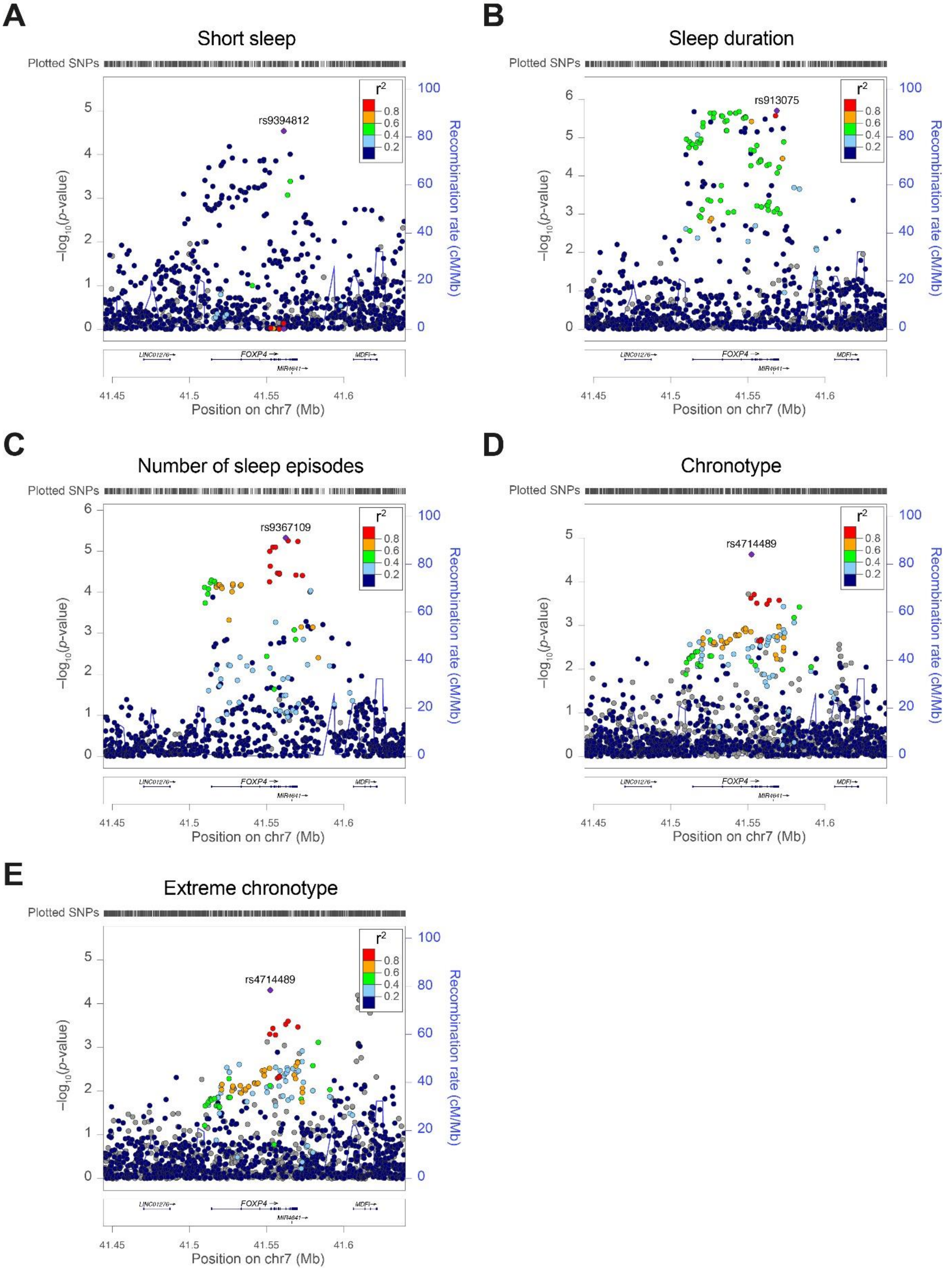

Supplemental Figure 3 – SNPs in *FOXP4* are associated with short sleep, sleep duration, number of sleep episodes, chronotype, and extreme chronotype. Regional association plots showing association signals for (**A**) short sleep, (**B**) sleep duration, (**C**) number of sleep

episodes, (**D**) chronotype, and (**E**) extreme chronotype at the *FOXP4* locus. Data are shown as $-\log_{10}$(*p*-value) for individual SNPs. The color of each marker reflects its linkage disequilibrium (r^2) with the strongest associated SNP indicated as a purple diamond. The recombination rate is indicated in blue. Chr, chromosome; cM, centimorgan; Mb, megabase.

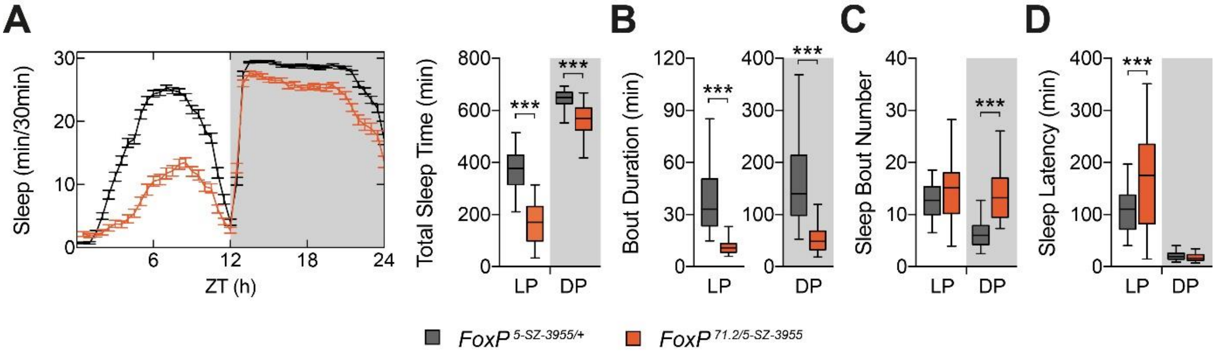

Supplemental Figure 4 – *FoxP* mutant females have short and fragmented sleep. (**A**) *FoxP*$^{71.2/5\text{-}SZ\text{-}3955}$ compound heterozygous mutant females flies (n = 76) show decreased total sleep time during both the day and the night ($p < 0.0001$) compared to heterozygous hypomorphic *Fox*$^{5\text{-}SZ\text{-}3955/+}$ flies (n = 97). (**B** and **C**) The average sleep bout duration of *FoxP*$^{71.2/5\text{-}SZ\text{-}3955}$ mutant flies is strongly reduced during both the day and the night ($p < 0.0001$), whilst the number of bouts is increased only during the night ($p < 0.0001$). (**D**) *FoxP*$^{71.2/5\text{-}SZ\text{-}3955}$ mutant female flies show increased latency to fall asleep after lights-on ($p = 0.00028$), but normal sleep onset latency to lights-off ($p = 0.23$). Data are represented as boxplots that extend from the 25th to the 75th percentiles, with the median indicated. Whiskers indicate the 5th and 95th percentiles. Statistical analysis was performed using two-tailed unpaired t-tests or Mann-Whitney tests, with Bonferroni correction for multiple comparisons. p-values are indicated as follows: ***$p < 0.001$.

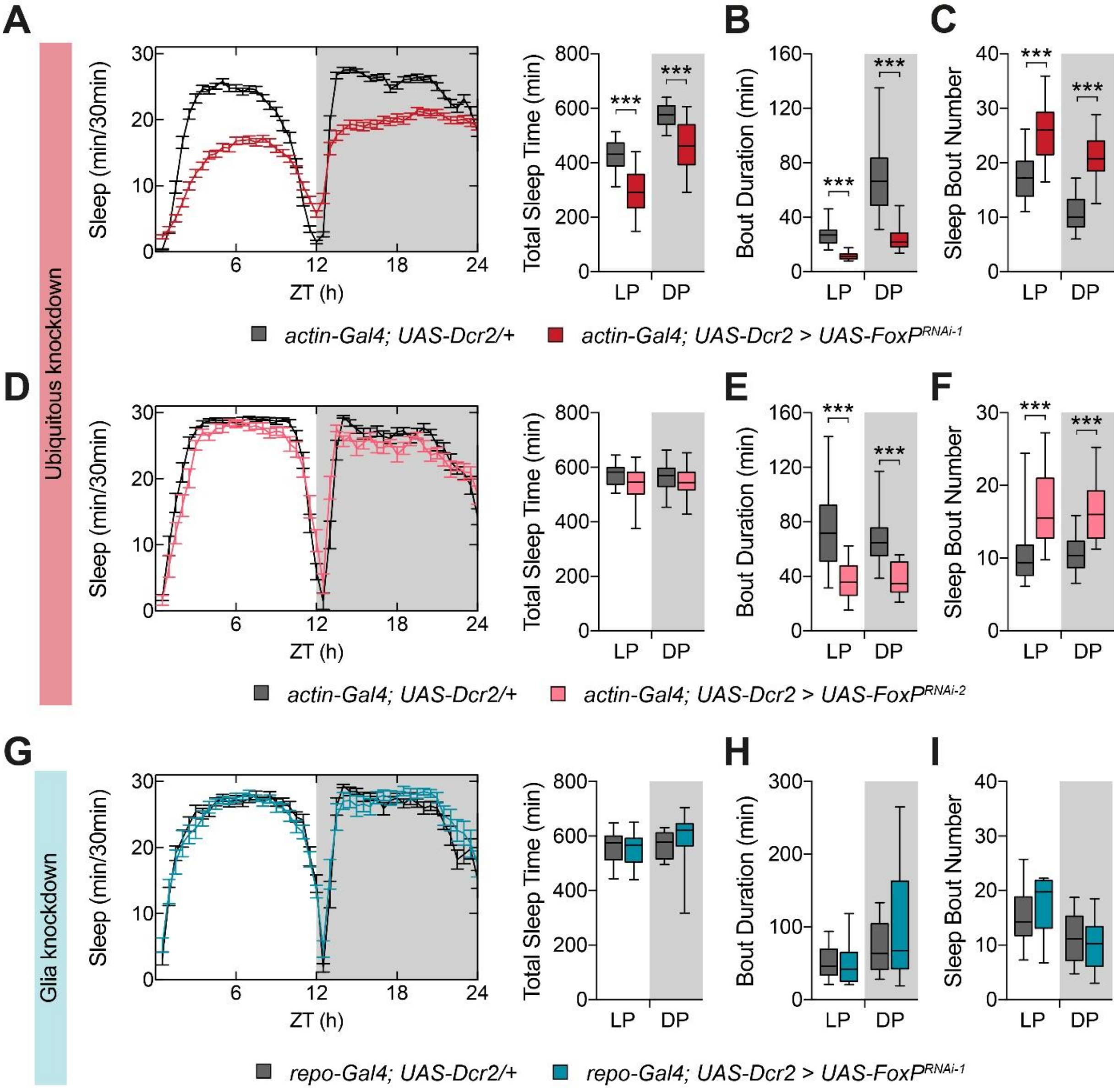

Supplemental Figure 5 – Ubiquitous *FoxP* knockdown, but not glia-specific, leads to decreased and fragmented sleep. (**A**, **D**, and **G**) Sleep profiles and their quantification. (**B**, **E**, and **H**) Average duration and (**C**, **F**, and **I**) number of their sleep bouts in the light (LP, ZT0-12) and dark periods (DP, ZT12-24). (**A-C**) Ubiquitous *FoxP* knockdown male flies (*actin-Gal4 > UAS-Dcr2, UAS-FoxP*$^{RNAi\text{-}1}$, n = 87) show a decrease in sleep duration during both the day and the night (p < 0.0001) compared to isogenic controls (*actin-Gal4, UAS-Dcr2/+*, n = 61). Moreover, ubiquitous knockdown flies show sleep fragmentation characterized by shorter but more frequent sleep bouts (p < 0.0001) during day and night. (**D-F**) Ubiquitous *FoxP* knockdown male flies with an independent RNAi (*actin-Gal4 > UAS-Dcr2, UAS-FoxP*$^{RNAi\text{-}2}$, n = 19) show sleep fragmentation during both the day and the night (p < 0.0001) when compared to isogenic controls (*actin-Gal4, UAS-Dcr2/+*, n = 22). (**G-I**) Pan-glial *FoxP* knockdown male flies (*repo-Gal4 > UAS-Dcr2, UAS-FoxP*$^{RNAi\text{-}1}$, n = 17) show no difference in sleep duration or

architecture compared to isogenic controls (*repo-Gal4, UAS-Dcr2/*+, $n = 18$). Data are represented as boxplots that extend from the 25th to 75th percentiles with the median indicated. Whiskers indicate the 5th and 95th percentiles. Statistical analysis was performed using two-tailed unpaired t-test or Mann-Whitney test, with Bonferroni correction for multiple comparisons. *p*-values are indicated as follows: ***$p < 0.001$.

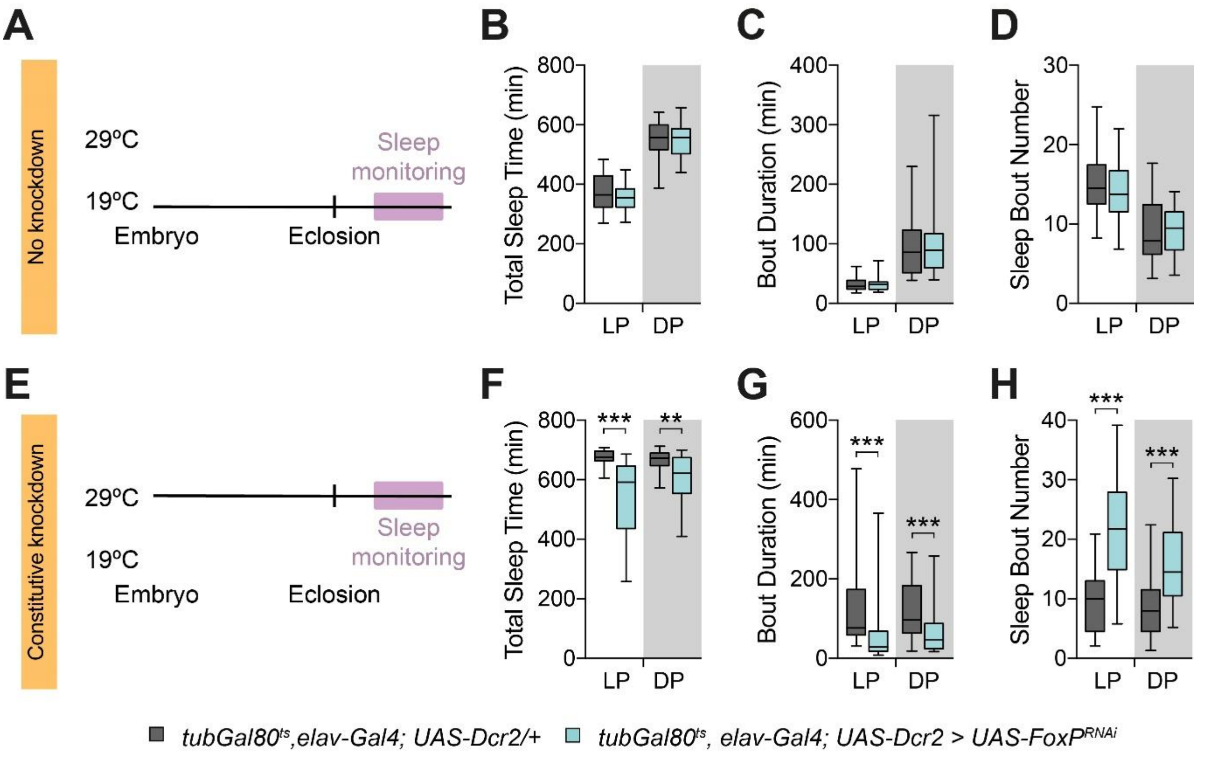

Supplemental Figure 6 – TARGET *FoxP* knockdown flies only show decreased and fragmented sleep when reared at permissive temperatures. (**A**) Temperature conditions used to not induce *FoxP* knockdown. (**B**) Representative data for total sleep time, (**C**) average sleep bout duration, and (**D**) number of sleep bouts during the light period (LP, ZT0-12) and dark period (DP, ZT12-24). Sleep amount and architecture remains unaltered in *tub-Gal80^{ts}, elav-Gal4; UAS-Dcr2 > UAS-FoxP^{RNAi-1}* flies (n = 31) reared and monitored at restrictive temperature (19°C) compared to background controls (*tub-Gal80^{ts}, elav-Gal4; UAS-Dcr2/+*, n = 32). (**E**) Temperature conditions used to induce constitutive *FoxP* knockdown. (**F**) Representative data for total sleep time, (**G**) average sleep bout duration, and (**H**) number of sleep bouts during the light period (LP, ZT0-12) and dark period (DP, ZT12-24). When reared and monitored at permissive temperature (29°C) *tub-Gal80^{ts}, elav-Gal4; UAS-Dcr2 > UAS-FoxP^{RNAi-1}* flies (n = 26) show decreased sleep in the LP (p < 0.0001) and the DP (p = 0.0029) compared to background controls (*tub-Gal80^{ts}, elav-Gal4; UAS-Dcr2/+*, n = 31). In these periods, knockdown flies have shorter sleep bouts (LP p < 0.0001; DP p = 0.0008) with an increase in their number (LP and DP p < 0.0001). Data are presented as boxplots showing the 25th to 75th percentiles, with the median indicated; whiskers represent the 5th and 95th percentiles. Statistical analysis was performed using two-tailed unpaired t-tests or Mann-Whitney tests, with Bonferroni correction for multiple comparisons. p-values are indicated as follows: **p < 0.01, ***p < 0.001.

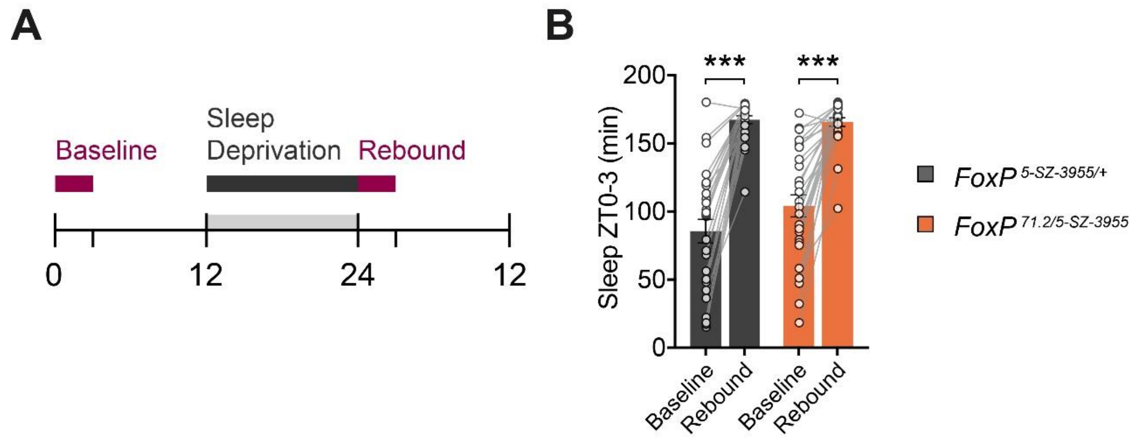

Supplemental Figure 7 – *FoxP* mutants have an intact sleep homeostat. (**A**) Schematic representation of our sleep deprivation regimen. (**B**) Sleep time duration of flies at ZT0-3 before (baseline) and after (recovery) 12 hours of mechanical sleep deprivation. Both heterozygous hypomorphic *Fox*$^{5\text{-}SZ\text{-}3955/+}$ flies (n = 27) and *FoxP*$^{71.2/5\text{-}SZ\text{-}3955}$ compound heterozygous male mutants (n = 27) showed significant rebound after 12 hours of mechanical sleep deprivation (both p < 0.0001). Two-tailed paired Students t-test. Data are represented as mean ± SEM, with individual values indicated. p-values are indicated as follows: ***p < 0.001.

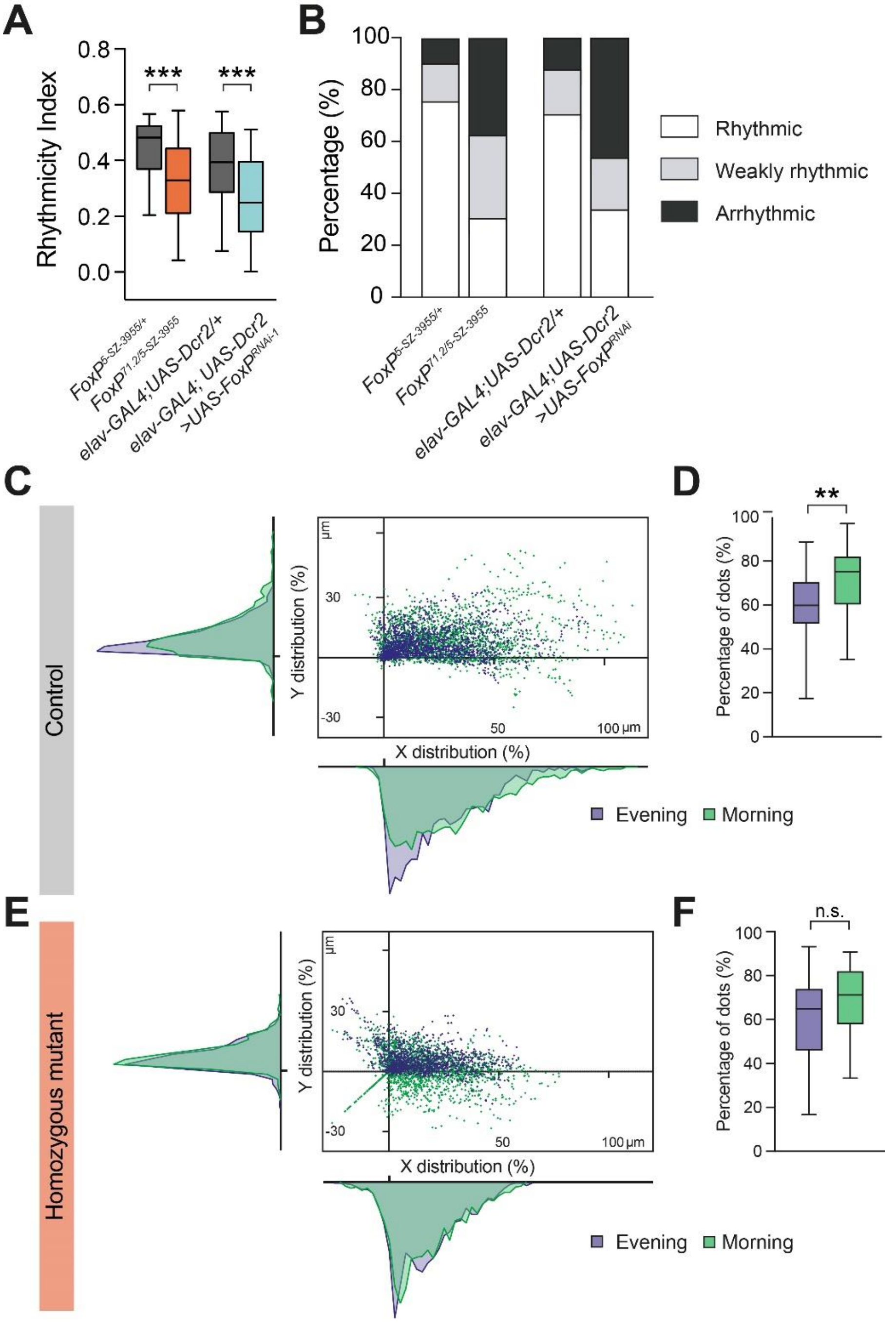

Supplemental Figure 8 – *FoxP* mutants show impaired circadian sLNv neuron plasticity. (**A**) Rhythmicity index and (**B**) proportion of rhythmic, weakly rhythmic, and arrhythmic flies in *FoxP*$^{71.2/5\text{-}SZ\text{-}3955}$ compound heterozygous mutant females (*n* = 74) compared to heterozygous hypomorphic *Fox*$^{5\text{-}SZ\text{-}3955/+}$ flies (*n* = 82), and pan-neuronal *FoxP* knockdown male flies (*UAS-Dcr2, elav-Gal4 > UAS-FoxP*^{RNAi}, *n* = 50) compared to isogenic controls (*UAS-Dcr2, elav-Gal4/+*, in grey, *n* = 58). *FoxP* transheterozygous mutants and *FoxP* pan-neuronal knockdowns

show increased arrhythmicity. (**C** and **E**) Distribution of Cartesian (X, Y)-coordinates for each individual PDF-immunoreactive maxima for the depicted genotypes. The accompanying distributions display the percentage of PDF-immunoreactive maxima along both the X and Y dimensions. (**D** and **F**) Percentage of PDF foci within the region that encompasses 50% of the PDF staining in the X dimension and 70% in the Y dimension in the controls of each genotype. (**C** and **D**) *FoxP* mutant isogenic controls ($FoxP^{+/+}$) flies show significant changes in s-LNvs axonal morphology between the morning (ZT1-3; in green, n = 28) and evening (ZT13-15; in blue, n = 25). (**E** and **F**) *FoxP* homozygous mutant ($FoxP^{71.2}$) flies do not show significant changes in s-LNvs axonal morphology between the morning (ZT1-3; in green n = 30) and evening (ZT13-15; in blue, n = 27). Boxplots represent median with 25-75th percentile and whiskers represent maximum and minimum. Significance was calculated using two-tailed unpaired t-test. p-values are indicated as follows: $^{**}p < 0.01$.

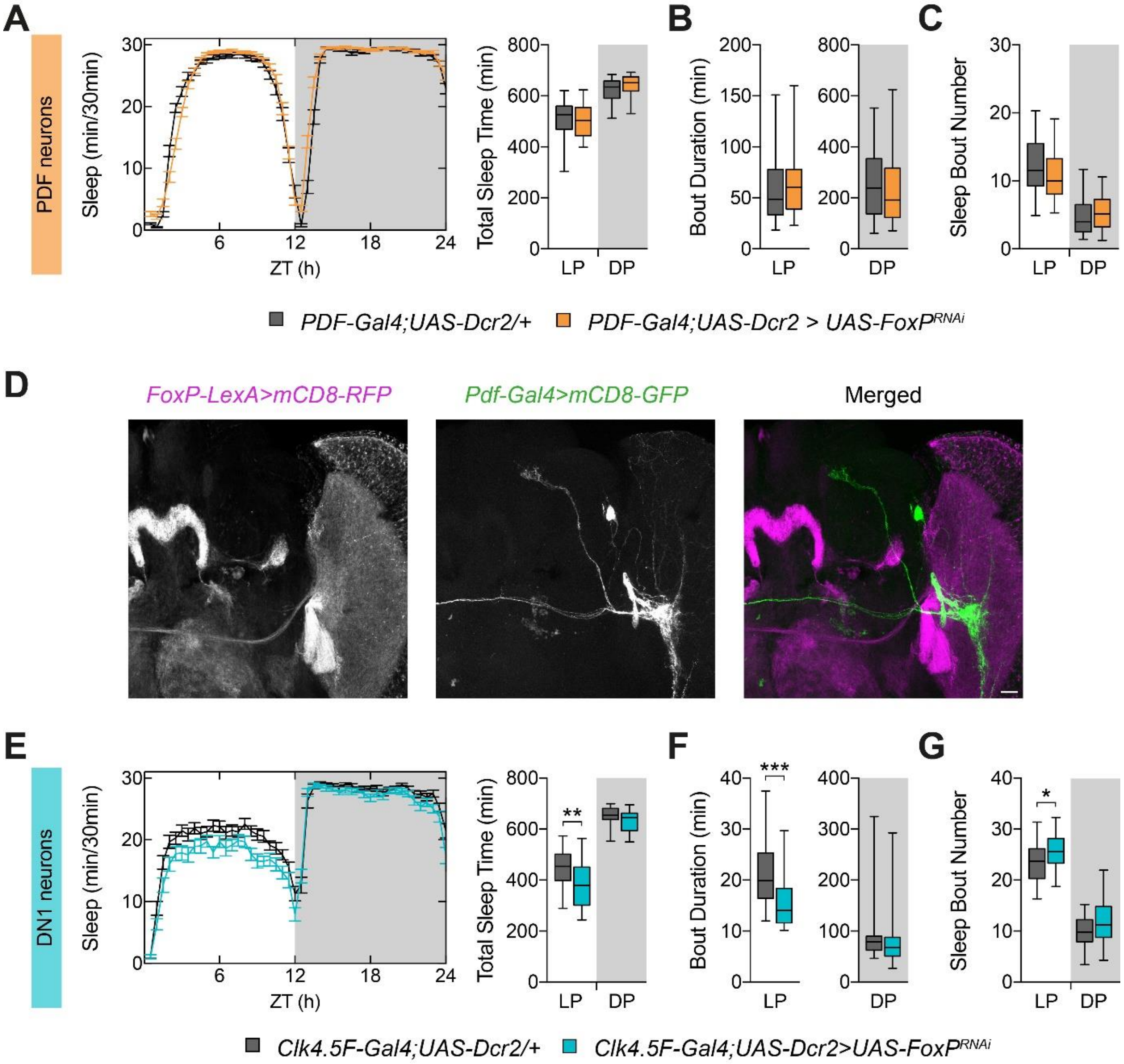

Supplemental Figure 9 – FoxP loss in DN1 neurons, but not in PDF neurons, alter sleep duration and architecture. (**A**) Total sleep time duration, (**B**) average duration and (**C**) number of their sleep bouts in the light period (LP, ZT0-12) and dark period (DP, ZT12-24). *FoxP* knockdown in PDF neurons (*PDF-Gal4, UAS-Dcr2* > *UAS-FoxP^{RNAi-1}*, *n* = 92) does not alter sleep duration nor architecture when compared to isogenic controls (*PDF-Gal4; UAS-Dcr2/+*, *n* = 71). (**D**) Colocalization of FoxP^{+} and PDF^{+} neurons; *FoxP-LexA* and *Pdf-Gal4* were combined with *LexOp-mCD8-RFP* and *UAS-mCD8-GFP*, respectively, to identify overlapping fluorescent signal in adult brains. FoxP is not expressed in PDF^{+} neurons in adults. Scale bar represents 25 µm. (**E**) Total sleep time duration, (**F**) average duration and (**G**) number of their sleep bouts during the LP and DP. *FoxP* knockdown in DN1 neurons (*Clk4.5F-Gal4, UAS-Dcr2*

> *UAS-*$FoxP^{RNAi-1}$, *n* = 28) leads to a decrease in daytime sleep (p = 0.0032) due to shorter sleep bouts (p = 0.0009) when compared to isogenic controls (*Clk4.5F-Gal4; UAS-Dcr2/*+, *n* = 28). These shorter bouts are accompanied by a slight but significant increase in their number (p = 0.045). Data are represented as boxplots that extend from the 25th to the 75th percentiles, with the median indicated. Whiskers indicate the 5th and 95th percentiles. Two-tailed unpaired t-test or Mann Whitney test, with Bonferroni correction for multiple testing. p -values are indicated as follows: *$p < 0.05$, **$p < 0.01$, ***$p < 0.001$.

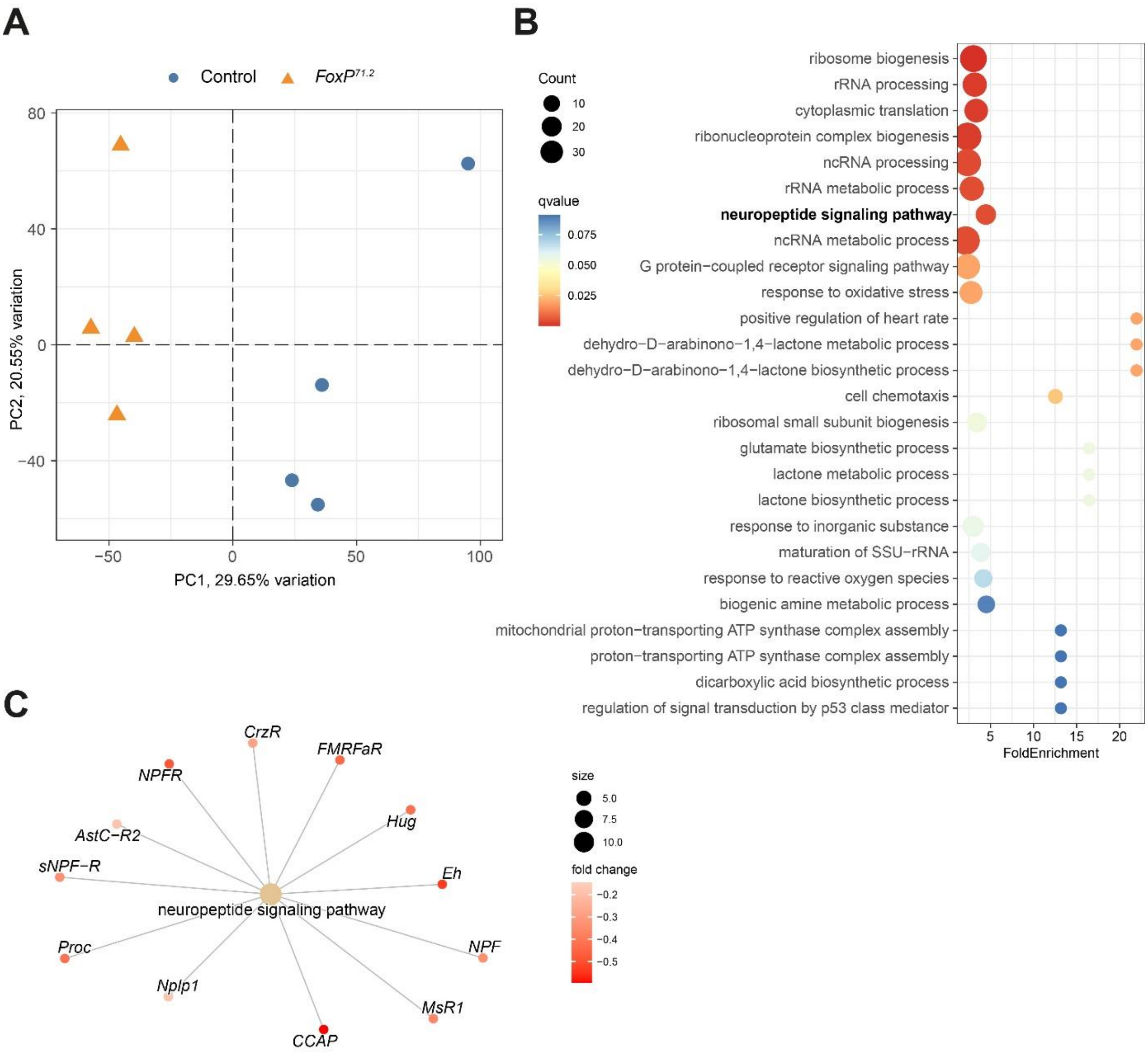

Supplemental Figure 10 – FoxP regulates genes enriched for neuropeptide signaling pathway. (**A**) Principal component analysis (PCA) of the regularized-log transformation of the normalized counts of all genes. (**B**) Dot-plot of GO biological process enrichment analysis from genes significantly down-regulated in the brains of *FoxP*$^{71.2}$ homozygous mutants. (**C**) Gene network illustrating significant genes associated with the biological process “neuropeptide signaling pathway”.

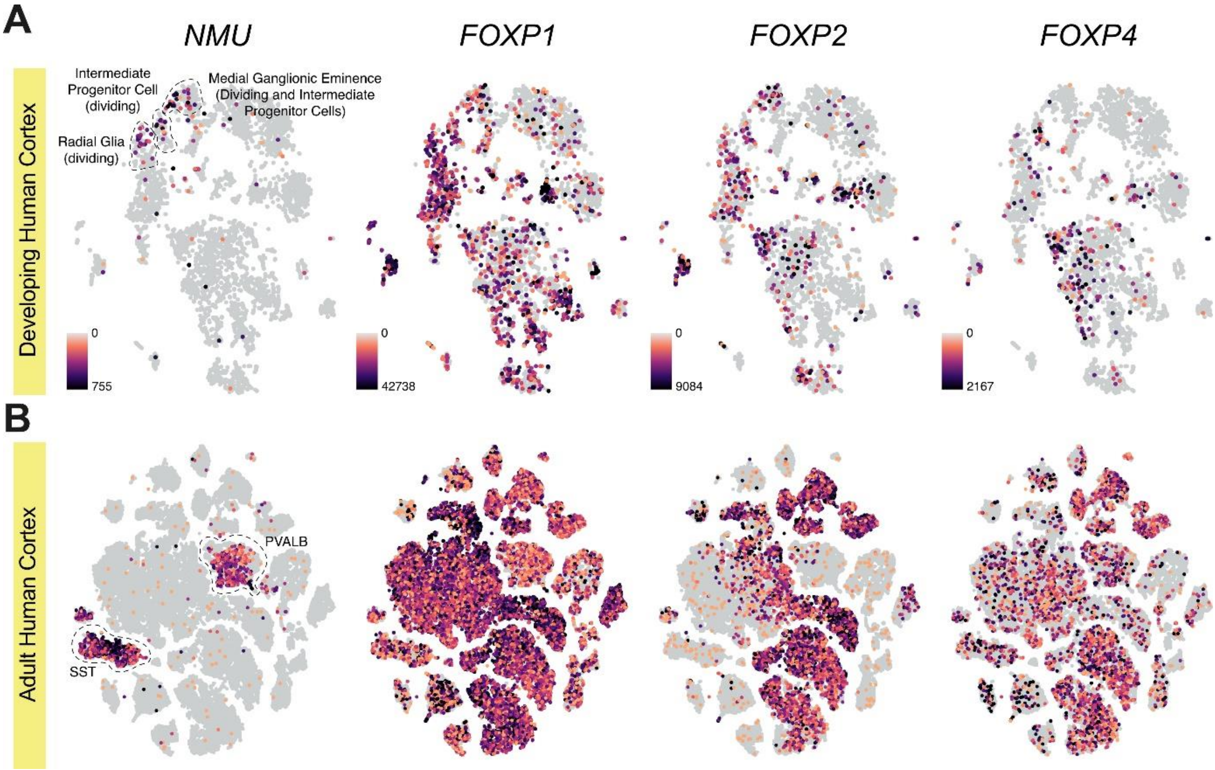

Supplemental Figure 11 – Human *FOXP1/2/4* are co-expressed with the mammalian counterpart of Hugin, *NMU*. (**A** and **B**) Expression of NMU and the different members of the FOXP gene family in single-cell RNA-sequencing data of both prenatal and adult human cortex. *FOXP1/2/4* are expressed in the same neurons that express the *Hugin* human homologue *NMU*. Scatterplots after principal components analysis and t-stochastic neighbor embedding (tSNE) from left to right: *NMU*, *FOXP1*, *FOXP2*, and *FOXP4* expression. SST, somatostatin; PVALB, parvalbumin-expressing neurons.
